# Associations between Recession hardships and subjective and objective sleep measures in the midlife in the United States study: race and gender differences

**DOI:** 10.3389/frsle.2024.1403818

**Published:** 2024-10-30

**Authors:** Aarti C. Bhat, Jose A. Diaz, Sun Ah Lee, David M. Almeida, Soomi Lee

**Affiliations:** ^1^Human Development and Family Studies, The Pennsylvania State University, University Park, PA, United States; ^2^Center for Healthy Aging, The Pennsylvania State University, University Park, PA, United States; ^3^Population Research Institute, The Pennsylvania State University, University Park, PA, United States

**Keywords:** Recession, stress, sleep, social determinants of health, midlife, actigraphy

## Abstract

**Objectives:**

This study investigates the associations of retrospective reports of Recession hardships with 10-year changes in subjective and objective indicators of sleep, and whether these associations differ by race and gender.

**Methods:**

Five hundred and one adults (14.57% Black; 54.49% female) from the Midlife in the United States (MIDUS) study reported on the subjective Pittsburgh Sleep Quality Index (PSQI) across two waves (pre-Recession, collected 2004–2009; post-Recession, collected 2017–2022), as well as Recession hardships since 2008. A sub-sample of 201 adults (25.37% Black; 58.21% female) provided objective actigraphy-measured sleep data (total sleep time, sleep onset latency, and sleep efficiency) across the two waves.

**Results:**

Descriptive analyses revealed Black participants had higher average Recession hardships, poorer post-Recession PSQI scores, and poorer post-Recession actigraphy sleep quantity and quality compared to white participants. Females had higher average Recession hardships compared to males; and reported poorer post-Recession PSQI, but had better objective post-Recession sleep quantity and quality compared to males. Regression models showed Recession hardships (across overall events, and sub-domains of financial and housing hardships) were associated with poorer PSQI and actigraphy-measured sleep efficiency following the Recession, adjusting for sociodemographic covariates, corresponding pre-Recession sleep variables, and pre-Recession chronic conditions. There was no evidence for significant moderation by race on sleep outcomes. However, gender moderation indicated associations between housing hardships and poorer actigraphy-measured sleep efficiency were more apparent for females than for males.

**Conclusions:**

Findings indicate that Recession hardships (particularly in financial and housing domains) may be manifested in poor sleep. Racial and gender groups may have differential exposure and sleep-related reactivity to Recession hardships.

## 1 Introduction

The Great Recession of 2007–2009 was the deepest and longest macroeconomic decline in United States (U.S.) history since the 1930's (Forbes and Krueger, 2019). The resulting economic hardships affected multiple life domains, including financial (median family income declined 8 and 25% of families lost at least 75% of their wealth), job (unemployment rose to 10%), and housing insecurity [over 15% of mortgages became delinquent or in foreclosure (Danziger, 2013; Pfeffer et al., 2013; Margerison-Zilko et al., 2016). These hardships are associated with sleep problems (Van Reeth et al., 2000; Epel et al., 2018; Lo Martire et al., 2020), including difficulties with sleep onset, maintenance, quality, and feeling unrested (Kalousová et al., 2019; Niekamp, 2019). Studies have also indicated that specific domains of economic hardship (examined outside of the context of Recession-specific hardships] can also contribute to sleep issues. Ongoing financial hardship is associated with lower sleep efficiency in late life (Hall et al., 2008); perceived job-related hardship is associated with poorer subjective sleep quality among midlife and older adults (Kim et al., 2021b); and housing hardship is associated with reductions in subjective sleep duration and quality (Liu et al., 2014; Bozick et al., 2021). However, this extant literature does not assess the potential impacts of multiple hardship domains within the same study on subjective and objective sleep indicators.

Recession hardships are representative of key hallmarks of stressors, including posing an immediate challenge while contributing to potentially persistent uncertainty; and can thus be classified as a chronic stressor with potential long-term health and wellbeing implications (Almeida et al., 2011, 2020, 2023). This classification of economic and recessionary hardships as crucial stressors ties back to foundational work by Glen H. Elder identifying economic hardships experienced during the Great Depression as critical persistent chronic stressors that can affect health, and likely important health behaviors such as sleep, across time (Elder, 2001). Recession and economic hardships may contribute to sleep problems through psychological pathways—specifically, mental health (Kalousová et al., 2019; Troxel et al., 2020). Recession and economic hardships have been shown to contribute to declines in mental health, including increased rates of depression, anxiety, rumination, hypervigilance, and suicide (Guastella and Moulds, 2007; Reeves et al., 2012; Hawton and Haw, 2013; McInerney et al., 2013; Cagney et al., 2014; Afifi et al., 2015; Wilkinson, 2016; Pruchno et al., 2017; Forbes and Krueger, 2019; de Bruijn and Antonides, 2020). Mental health issues, including depression and rumination, are linked to poorer sleep quality and quantity across subjective and objective indicators, including the Pittsburgh Sleep Quality Index (PSQI) subjective sleep measure, and objectively measured total sleep time, sleep onset latency, and sleep efficiency (Guastella and Moulds, 2007; Anderson and Bradley, 2013; Pillai et al., 2014; Steiger and Pawlowski, 2019; Clancy et al., 2020; Palagini et al., 2022). Mental health decline and stress can activate the hypothalamic-pituitary-adrenal (HPA) axis, which plays a critical role in sleep-wake cycle regulation (Van Reeth et al., 2000). Dysregulation of the HPA axis contributes to fragmented and unstable sleep patterns, disruption of sleep homeostasis, and sleep disorders (Van Reeth et al., 2000; Lo Martire et al., 2020). Crucially, this relationship between mental health and sleep is bidirectional, such that mental health issues may contribute to sleep issues, which in turn further exacerbate mental health decline (Fang et al., 2019). Stress process models (Pearlin et al., 1981; Epel et al., 2018) illustrate the complex biopsychosocial pathways by which chronic stressors such as disruptive economic hardships can lead to psychological, behavioral, physical, and physiological health outcomes, and support the interdependence of these mechanisms. Thus, sleep may be one pathway that contributes to mental, physical, and biological health disparities in the context of Recession hardships (Forbes and Krueger, 2019; Patel, 2019; Foverskov et al., 2020; Bhat et al., 2022).

Poor sleep (both quality and quantity) increases risk of developing and exacerbating health conditions, including cardiometabolic diseases, cancer, cognitive issues, inflammation, accelerated biological aging, dementia, and mortality (Luyster et al., 2012; Pigeon et al., 2012; Kim et al., 2016; Rangaraj and Knutson, 2016; Chirinos et al., 2017). This is particularly so for aging adults, with research indicating “sleep may be as important to health in old age as diet and exercise” (Scommegna, 2018, p. 1). Given the rapidly aging U.S. population (Preston and Vierboom, 2021) and increasing economic strain faced by aging adults, it is important to examine change in sleep associated with exposure to macro-level economic stressors in the aging population (Sheehan et al., 2019; Bierman, 2021). In this study, we use stressful experiences encountered due to the unique historical context of the Great Recession as an example of macro-level economic stressors and assess their associations with changes in sleep quantity and quality.

The social determinants of health (SDoH) perspective (Navarro, 2009) suggests that political, economic, social, and environmental contexts contribute to health inequalities between groups in a population, including between racial and gender groups. Demographic characteristics may contribute to increased vulnerability to Recession-induced hardship exposure and resulting deleterious sleep health consequences. Relative to the white population, racial/ethnic minorities (particularly Black and Hispanic groups) have elevated levels of: financial hardship, including lesser intergenerational wealth and incomes (Pfeffer et al., 2013); job hardship, including higher unemployment rates and limited upward career mobility opportunities (Borkowski et al., 2024); and housing hardship, including lower homeownership and higher eviction rates (Kuebler, 2013; Sharp et al., 2020). Many of these disadvantages were exacerbated by the Recession, including wealth inequality, disproportionate unemployment rates, and higher foreclosure rates for the Black population in the U.S. compared to the non-Hispanic white population; and these impacts have been long-lasting for the economic recovery of the Black population (Engemann and Wall, 2009; Houle, 2014; Addo and Darity, 2021). Additionally, Black adults report poorer sleep, have shorter sleep duration, longer sleep onset latency, and higher rates of waking after sleep onset (measured by actigraphy) relative to non-Hispanic white adults (Adenekan et al., 2013; Gamaldo et al., 2014; Fuller-Rowell et al., 2016; Grandner et al., 2016; Hale et al., 2020). These associations between race and poor subjective and objective measures of sleep may be partly attributable to experiences of discrimination and neighborhood disadvantage (e.g., living in lower socioeconomic status neighborhoods, with lesser access to resources and services such as greenspace, healthy foods, and healthcare, and increased exposure to neighborhood violence) that have been found to be more prevalent among Black adults compared to white adults (Adenekan et al., 2013; Fuller-Rowell et al., 2016; Grandner et al., 2016; Petrov and Lichstein, 2016; Ong et al., 2017; Owens et al., 2017). In fact, a study by Kirsch et al. (2019) found that poor neighborhood environment was found to be linked with poor sleep quality for Black adults. Poorly functioning cooling/heating systems, toxic exposures in the neighborhood, crowdedness, and noise pollution are all aspects of neighborhood disadvantage that minoritized groups are more exposed to, which may contribute to poorer sleep health (Adamkiewicz et al., 2011; Baker et al., 2017; Swope and Hernández, 2019; Mansour et al., 2022). Given that minoritized or marginalized adults are disproportionately exposed to oppression and experience the double jeopardy of socioeconomic status deprivation alongside discrimination according to the minority poverty hypothesis (Ong et al., 2017; Swope and Hernández, 2019; Surachman et al., 2020), as well as evidence that Black adults are more vulnerable to increases in chronic physical conditions when experiencing housing hardships compared to white adults (Bhat et al., 2022), it is possible that the convergence of systemic discrimination and disadvantage alongside Recession hardships (which may exacerbate already existing disadvantages) may lead to the proliferation of stressors and a cumulative, compounding, or exponential impact on stressor reactivity due to multiple stressor exposures (as theorized by stress process models), which could contribute to exacerbated sleep issues for Black adults (Almeida et al., 2011; McLeod, 2012; Pearlin and Bierman, 2013; Ong et al., 2017; Epel et al., 2018; Swope and Hernández, 2019; Almeida et al., 2020; Yip et al., 2021). In fact, in the aftermath of the Recession, Black adults reported marked declines in sleep duration compared to non-Hispanic whites (Sheehan et al., 2019), but the effects of Recession hardships on objective indicators of sleep between racial groups are yet unclear.

Gender may also be a factor in Recession hardship exposure and sleep quality/duration. While men experienced more rapid rises in unemployment than women during the Recession, post-Recession, men saw more rapid employment gains than women (Albelda, 2013). Additionally, reduced revenue and budget cuts due to the Recession have more disproportionately impacted women than men (Albelda, 2013). In terms of sleep, women have better objective sleep than men (longer sleep times, shorter sleep-onset latency, higher sleep efficiency), although these objective sleep advantages may narrow in older adulthood (Dzaja et al., 2005; Krishnan and Collop, 2006; Burgard and Ailshire, 2013; Mallampalli and Carter, 2014; Guidozzi, 2015). However, women report more sleep complaints, including poorer sleep quality measured by the Pittsburgh Sleep Quality Index (PSQI; Li et al., 2019) and insomnia symptoms, than men (the gender divergence for insomnia-related issues increases in older age; Dzaja et al., 2005; Krishnan and Collop, 2006; Guidozzi, 2015). Given that women have been shown to experience more mental health issues when facing economic stress than men, it is possible that, given the intertwined relationship between mental health and sleep, Recession hardships contribute to greater subjective and objective sleep issues for women as compared to men (Glonti et al., 2015).

While prior literature indicates an association between economic and Recession hardships and sleep problems, providing a greater understanding of how these types of stressful experiences can affect the crucial health behavior of sleep (Hall et al., 2008; Kalousová et al., 2019; Niekamp, 2019; Bierman, 2021; Kim et al., 2021b), these studies are limited by use of cross-sectional data on sleep; minimal to no recognition of differential sleep impacts of different domains of Recession hardships; lack of variety of sleep measures (mostly self-reports only); and lack of research examining differences by race and gender in the Recession-sleep link. These limitations are important in that, without controlling for sleep at a pre-Recession time point, it is difficult to elucidate whether Recession hardships may be a contributing factor to post-Recession sleep issues. Cross-sectional studies may indicate that Recession hardships and post-Recession sleep are correlated, but without use of longitudinal data, those who have sleep issues in a cross-sectional study may be simply more susceptible to economic and Recession hardships due to less energy and mental or physical resources to work and attain economic security. As studies have shown, healthier people tend to be more able to build financial security and wealth than unhealthy people (Adams et al., 2003). Thus, using longitudinal data may help with assessing change over time in sleep and potential directionality of the association between Recession hardships and sleep. Using different domains of Recession hardships within the same study can elucidate whether the effects on sleep are comparable across domains, or whether certain forms of hardship may be more predictive of sleep-related declines than others, which can provide information on what types of economic interventions may be most effective in protecting against declines in sleep health. Use of self-reports of sleep is valuable in assessing subjective experiences, but as we have previously discussed, for some groups subjective and objective indicators of sleep do not always align (Dzaja et al., 2005; Jackson et al., 2018, 2020). Thus, a strength of assessing both subjective and objective sleep measures in context of Recession hardships provides information on whether these indicators are congruent when experiencing economic stress, or whether the gap between perceptions and objective sleep measures may increase in context of Recession stress (Jackowska et al., 2011). Finally, assessing race and gender differences in sleep in the historic context of the Great Recession among a diverse sample can provide information on whether certain groups are more susceptible to the adverse sleep implications of Recession hardships, which may increase population health disparities. This is valuable information for targeted sleep and health interventions in the context of current and future economic and recessionary shocks.

To address limitations of prior work, we use longitudinal data of subjective and objective sleep measures, assess different Recession hardship domains, and examine race/gender moderation effects. Using a national sample of midlife and older adults, we address the following questions:

1. How are Recession hardships and subjective and objective sleep indicators distributed across the aging population?
2. Are Recession hardships negatively associated with subjective and objective sleep following the Recession?
3. Are specific types of Recession hardships (financial, job-related, and housing) differentially associated with sleep outcomes for midlife and aging adults?
4. Do race and gender interact with Recession hardships to relate to sleep outcomes?

Based on prior literature, we hypothesize that Recession hardships will be unevenly distributed such that Black and female participants will be more susceptible to exposure, respectively (Albelda, 2013; Thomas et al., 2017; Addo and Darity, 2021); that Black participants will have poorer subjective and objective sleep outcomes compared to white participants, while female participants will have poorer subjective but better objective sleep outcomes compared to male participants (Krishnan and Collop, 2006; Mallampalli and Carter, 2014; Guidozzi, 2015; Jackson et al., 2018, 2020); that Recession hardships across all domains will be associated with poorer subjective and objective indicators of sleep (Kalousová et al., 2019; Niekamp, 2019; Bozick et al., 2021; Kim et al., 2021b); that the null hypothesis that the individual domains of Recession hardships will have a fairly equivalent association on sleep outcomes will hold given no empirical evidence thus far comparing these domains to indicate the contrary; and that Black and female participants will be more susceptible to sleep issues when facing Recession hardships compared to white and male participants, respectively (Glonti et al., 2015; Bhat et al., 2022).

## 2 Methods

### 2.1 Participants

This study utilizes publicly available data from Midlife in the United States (MIDUS), a national longitudinal study of health and wellbeing, to assess effects of Recession hardships on various aspects of sleep health (https://midus.colectica.org/). MIDUS data collection is reviewed and approved by the Education and Social/Behavioral Sciences and the Health Sciences IRBs at the University of Wisconsin-Madison, and participants provided informed consent to participate in the study (Barry, 2014). This paper uses data from MIDUS 2 Survey (collected 2004–2006), Milwaukee Survey (introduced an oversample of African American adults to increase dataset diversity; collected 2005–2006), and Biomarker (collected 2004–2009) Projects; as well as from MIDUS 3 Survey (collected 2013–2014), Milwaukee Survey (collected 2016–2017), and Biomarker (collected 2017–2022) Projects (to see a timeline of data collection with sample sizes across MIDUS waves, reference: https://midus.wisc.edu/data/timeline.php). The MIDUS 2 and 3 Biomarker Projects collected data from a subsample of participants in the MIDUS 2 and 3 Survey Projects. In our analyses, MIDUS 2 (referred to as M2 in tables) serves as a pre-Recession assessment and MIDUS 3 (referred to as M3 in tables) is a post-Recession assessment conducted ~10 years later.

While the MIDUS 1 (the first wave of MIDUS) Survey Project (collected 1995–1996) had 7,108 participants, the MIDUS 2 Survey Projects (including participants from the main longitudinal sample and the Milwaukee oversample introduced at MIDUS 2) had 5,555 participants, and the MIDUS 3 Survey Projects (including participants from the main longitudinal sample and the Milwaukee oversample) had 3,683 participants, indicating attrition between waves. Given that this study utilizes the MIDUS 2 and 3 waves, only 3,683 participants had data from both the MIDUS 2 and MIDUS 3 Survey Projects. The PSQI and actigraphy-measured sleep scores were collected in the MIDUS 2 and MIDUS 3 Biomarker Projects (which had 1,255 and 747 participants, respectively). Sixty participants who participated in the MIDUS 3 Biomarker Project did not participate in the MIDUS 2 Biomarker project (which provided baseline sleep measures); thus there were only 687 participants who had data from both the MIDUS 2 and MIDUS 3 Biomarker Projects and were eligible for inclusion in the final samples. Supplementary Table 1 describes sample attrition between pre-Recession (MIDUS 2) and post-Recession (MIDUS 3) Biomarker Projects. Of the sample of 687 participants, only those with complete data on post-Recession sociodemographic characteristics (and who identified race as white or Black), pre-Recession chronic conditions, and sleep measures of interest at pre- and post-Recession timepoints (which were provided in the MIDUS 2 and 3 Biomarker Projects) were included. Supplementary Table 2 illustrates the results of these sociodemographic and health conditions selection criteria among the sample of 687 participants, which resulted in our final sample size for the main analytic sample. The main analytic sample size was *N* = 501 (14.57% Black, 54.49% female). Participants in the actigraphy sample had at least 3 out of 7 days of Actiwatch data; sample size was *N* = 201 (25.37% Black, 58.21% female). Table 1 provides descriptives of pre-Recession and post-Recession variables for both the main analytic and actigraphy samples.

**Table 1:** Descriptive statistics for main analytic sample (*N* = 501) and actigraphy sample (*N* = 201), MIDUS 2 and 3.

**Variable**	**Main analytic sample (*****N*** = **501)**				**Actigraphy sample (*****N*** = **201)**			
	**%**	**Mean**	**SD**	**Range**	**%**	**Mean**	**SD**	**Range**
**Recession hardships (%** = **1**+ **hardships; M3)**								
Overall Recession hardships	77.45	2.43	2.67	0–13	77.11	2.53	2.73	0–13
Financial hardships	70.46	1.37	1.30	0–6	71.14	1.41	1.32	0–5
Job-related hardships	31.34	0.54	0.97	0–4	29.35	0.51	0.96	0–4
Housing hardships	27.54	0.51	1.04	0–6	32.84	0.61	1.09	0–5
**Sociodemographic variables (M3)**								
**Race**								
White	85.43				74.63			
Black	14.57				25.37			
**Gender**								
Male	45.51				41.79			
Female	54.49				58.21			
Age		65.60	9.46	48-94		64.91	9.50	48-92
**Marital status**								
Not married	39.52				41.29			
Married	60.48				58.71			
**Highest educational level**								
High school or less	20.56				27.36			
Some college or more	79.44				72.64			
**Recession change in income**								
About same	27.74				28.36			
Less currently	33.73				34.83			
More currently	38.52				36.82			
**Sociodemographic variables (M2)**								
Age		53.54	9.34	35–82		52.84	9.38	35–82
**Marital status**								
Not married	31.74				33.33			
Married	68.26				66.67			
**Highest educational level**								
High school or less	21.76				25.87			
Some college or more	78.24				74.13			
Total household income		78,545.51	63,111.44	0–3 x 10^5^		69,493.34	56,509.07	0–3 x 10^5^
**Health variables**								
Chronic conditions (% = 1+ conditions; M2)	77.84	1.90	1.67	0–10	79.10	1.99	1.84	0–10
Global PSQI (M2)		5.99	3.50	1–19				
≤ 5	54.49							
>5	45.51							
Global PSQI (M3)		6.21	3.44	0–18				
≤ 5	50.70							
>5	49.30							
Total sleep time (M2)						369.27	65.26	161.14–615.92
Total sleep time (M3)						397.53	67.12	166.93–575.38
Sleep onset latency (M2)						28.46	24.38	0.21–174.86
Sleep onset latency (M3)						22.75	20.19	1.86–112.50
Sleep efficiency (M2)						80.03	9.67	39.94–93.61
Sleep efficiency (M3)						83.86	8.08	56.10–95.82

### 2.2 Measures

#### 2.2.1 Recession hardships

The MIDUS 3 Survey asked participants a set of yes/no questions regarding events experienced during and in the aftermath of the 2007–2009 Great Recession across domains of financial (e.g., increased debt), job (e.g., lost job), and housing impacts (e.g., lost home to foreclosure). We included 18 Recession hardship items, calculating number of experiences in each domain as well as across domains (following operationalization of Forbes and Krueger, 2019). Supplementary Table 3 provides prevalence of hardship domains in the main analytic sample, broken down by Recession item as well as by race and gender.

#### 2.2.2 Pittsburgh Sleep Quality Index

In MIDUS 2 and MIDUS 3 Biomarker Projects, participants responded to 19 items that assessed sleep experiences across seven sub-components during the past month. Each item is scored as categorical values ranging from 0 to 3 (e.g., 0 = not during the past month; 1 = less than once a week; 2 = once or twice a week; 3 = three or more times per week; Buysse et al., 1989; Cox et al., 2022). The seven sub-components were: (1) subjective sleep quality (e.g., “how would you rate your sleep quality overall?”), (2) sleep onset latency (e.g., “how long has it taken you to fall asleep at night?”), (3) sleep duration (e.g., “how many hours of actual sleep did you get at night?”), (4) habitual sleep efficiency (e.g., “when have you usually gone to bed at night?”), (5) sleep disturbance (e.g., “how often have you had trouble sleeping because you woke up in the middle of the night or early morning?”), (6) use of sleeping medication (e.g., “how often have you taken medicine to help you sleep?”), and (7) daytime dysfunction (e.g., “how often have you had trouble staying awake while driving, eating meals, or engaging in social activity?”; Buysse et al., 1989; Li et al., 2019). The PSQI Global Sleep Score was constructed by summing the seven sleep components for each participant with complete data (for more detailed information about the PSQI see https://www.sleep.pitt.edu/instruments/). Across all components with the exception of daytime dysfunction, adults 65 years and older generally have higher scores compared to younger adults (Kim et al., 2021a). The post-Recession PSQI global score was used as an outcome in the analysis; while the pre-Recession PSQI global score was utilized as a covariate. A Cronbach's alpha test of the post-Recession PSQI global score indicated a reliability score of 0.71 (95% CI = 0.67, 0.75). Supplementary Table 4 illustrates the mean of post-Recession PSQI scores across the main analytic sample and by race and gender.

#### 2.2.3 Sleep actigraphy measures

In MIDUS 2 and MIDUS 3 Biomarker Projects, a subgroup of participants wore Mini Mitter Actiwatch^®^-64 activity monitors on their non-dominant wrist for seven consecutive days and nights, simultaneously completing daily diaries indicating bedtime and wake time, which were used to cross-check start and end times for actigraphy records (Dienberg Love et al., 2010; Lemola et al., 2013; Kim et al., 2016; Brindle et al., 2019). For this study, the following actigraphy measures were used:

1. *Total sleep time*, defined as the total amount of minutes scored as sleep in a given diary-defined interval.
2. *Sleep onset latency*, defined as the duration from bedtime to sleep onset in minutes.
3. *Sleep efficiency*, calculated as the percentage of time spent asleep to total time spent in bed, multiplied by 100.

Total sleep time, sleep onset latency, and sleep efficiency are commonly used actigraphy sleep measures in studies using MIDUS data (e.g., Kim et al., 2016; Chung, 2017; Owens et al., 2017; Yip et al., 2021), and provide information regarding aspects of sleep quantity (total sleep time) and quality (sleep onset latency, sleep efficiency). According to the National Sleep Foundation, older adults should, ideally, be getting between 7 and 8 h of total sleep time per night, and sleep durations that are either too short or excessively long are associated with poorer mental and physical health (Chaput et al., 2018). Total sleep time tends to decline with aging (Espiritu, 2008; Li et al., 2018). Sleep onset latency is considered a quantity that provides information on quality of sleep (Littner et al., 2005; Jung et al., 2013; Shrivastava et al., 2014). On average, sleep onset latency is between 10 and 20 min for healthy adults; sleep onset latency of < 8 min may be indicative of sleep deprivation or a sleeping disorder such as narcolepsy (Littner et al., 2005); whereas sleep onset latency of more than 20 min could be indicative of issues such as insomnia (Gandhi et al., 2021). Sleep onset latency tends to increase with aging (Moraes et al., 2014). High sleep onset latency may contribute to sleep debt, or how much sleep one has missed, which may accumulate across multiple nights, adversely affecting both physical wellbeing and cognitive abilities (Spiegel et al., 1999; Ancoli-Israel, 2009; Banks et al., 2010). Total sleep time and sleep onset latency are among factors (along with waking after sleep onset) that affect sleep efficiency (Reed and Sacco, 2016). Across age groups, the National Sleep Foundation judges that sleep efficiency ≥85% indicates good sleep quality, whereas sleep efficiency ≤ 74% is indicative of poor sleep quality (Ohayon et al., 2017). Sleep efficiency does generally decrease among older adults, and lower sleep efficiency is associated with higher prevalence of depression, hypertension, circulatory problems, cardiovascular disease, arthritis, and breathing issues among older adults (Didikoglu et al., 2020; Yan et al., 2021).

Each of these measures at post-Recession was aggregated as a mean score across the number of days participants wore the watch and utilized as outcomes in analyses; while the corresponding pre-Recession actigraphy mean scores were covariates in analyses. Across the actigraphy sample (*N* = 201), 94.53% of participants at pre-Recession assessment wore the watch all 7 days (Range = 3–7); while 92.54% of participants at post-Recession assessment wore the watch all 7 days (Range = 4–7). Supplementary Table 4 presents the mean of post-Recession sleep actigraphy measures across the actigraphy sample, as well as broken down by race and gender.

#### 2.2.4 Sociodemographic and health covariates

All covariates used in analyses were measured at post-Recession Survey assessment, except for age and marital status which were provided at post-Recession Biomarker assessment, and chronic conditions which were measured at pre-Recession Survey assessment (and pre-Recession PSQI and actigraphy sleep from the Biomarker assessment were also used as covariates in analyses).

##### 2.2.4.1 Race

Participants indicated racial background as White, Black/African American, Native American or Alaska Native Aleutian Islander/Eskimo, Asian, Native Hawaiian or Pacific Islander, or other. Participants who did not indicate their racial origins as white (reference group) or Black were excluded (7.42% of the original sample). Given limited representation of other racial groups in the MIDUS study, many other papers using MIDUS data have excluded participants who do not indicate race as white or Black when examining racial group differences (Fuller-Rowell et al., 2016; Surachman et al., 2020; Bhat et al., 2022).

##### 2.2.4.2 Gender

Participants indicated their gender as either male (reference group) or female. MIDUS only allows participants to self-classify as male or female, so the study fails to capture the spectrum of gender identities that exist (Heidari et al., 2016).

##### 2.2.4.3 Other sociodemographics

Standard sociodemographic variables were utilized as covariates in this study, in line with several MIDUS papers assessing health and sleep outcomes (e.g., Lemola et al., 2013; Chung, 2017; Stephan et al., 2017; Bhat et al., 2022). Age was treated as a continuous variable in the analyses. Other sociodemographics, such as marital status and education, were dichotomized, which is a common and often considered statistically appropriate practice for categorical covariates that are not main independent or dependent variables in analyses (Gustafson and Le, 2002; Li et al., 2023). Participants reported marital status as married, separated, divorced, widowed, never married, or living with someone in a committed relationship. MIDUS has fairly low percentages of participants reporting marital statuses other than married (in the main analytic sample, 1.60% report being separated, 15.17% report being divorced, 10.98% report being widowed, 11.38% report never being married, and 0.40% report living with someone). Thus, marital status is usually dichotomized when treated as a covariate and not a main variable of interest in analyses using the MIDUS study (e.g., Friedman and Herd, 2010; Lemola et al., 2013) to compare differences in outcomes by non-married (reference group) vs. married participants, which was the standard followed by this paper. Additionally, given that marriage provides certain taxation and financial benefits in the U.S., and that cohabitation has been found to be much less protective for health compared to marriage among aging adults in the U.S., there is further justification for dichotomizing the variable in this manner (Perelli-Harris et al., 2018). Participants reported their highest level of education completed as 1 = no school or some grade school (0.00%), 2 = junior high school (0.20%), 3 = some high school (2.99%), 4 = GED (1.00%), 5 = graduated from high school (16.37%), 6 = 1–2 years of college, no degree (17.96%), 7 = 3+ years of college, no degree (3.39%), 8 = graduated from 2 year vocational school or associate's degree (10.78%), 9 = graduated from 4 to 5 year college or bachelor's degree (23.95%), 10 = some graduate school (2.99%), 11 = master's degree (15.37%), and 12 = doctoral or other professional degree (4.99%; all percentages from main analytic sample). Dichotomizing highest education level into high school or less (reference group) and some college or more is common in papers utilizing MIDUS data, particularly when education is not the primary predictor/dependent variable being assessed, in order to assess group comparisons in analyses (e.g., Lemola et al., 2013; Wardecker et al., 2019; Bhat et al., 2022), and this study followed this standard. Additionally, studies have indicated that there are notable differences in certain health outcomes, such as depressive symptoms, between U.S. adults with a high school or less education, compared to those with some college or more; providing further rationale that dichotomizing education in this manner may be appropriate when used as a predictor/covariate for health outcomes (Mcfarland and Wagner, 2015). Participants described total household income compared to before the Recession as less currently, about the same (reference group), and more currently.

##### 2.2.4.4 Chronic conditions

Chronic conditions were reported by participants at pre-Recession assessment, by identifying whether or not they experienced 14 categories of chronic conditions over the past 12 months. Participants also indicated whether they had ever experienced cancer or heart disease, and these conditions were added to the number of chronic conditions score [following operationalization used by Piazza et al. (2013), and including anxiety/depression as an additional condition]. Chronic conditions at pre-Recession assessment were included as a covariate because controlling for prior health conditions may help elucidate whether sleep problems experienced at post-Recession assessment are related to simply having more health issues previously, rather than exposures that occurred after the pre-Recession assessment, such as Recession hardships. Sleep issues and chronic mental and physical conditions are highly intertwined, with research indicating a potential bidirectional relationship with sleep contributing to mental distress and multisystem biological dysregulation, thus contributing to or exacerbating chronic conditions, and chronic conditions contributing to more disrupted sleep among aging adults (Foley et al., 2004; Garcia, 2008; Carroll et al., 2015; Chai et al., 2020). Thus, pre-Recession chronic conditions serve as a crucial control to assess whether any variation in sleep issues at post-Recession assessment is not simply due to health selection effects (Hoffmann et al., 2019). Supplementary Table 5 illustrates the categories and prevalence of pre-Recession chronic conditions in the main analytic sample.

Supplementary Table 6 illustrates correlations between variables in the main analytic sample. Supplementary Table 7 provides univariable associations between Recession hardships, race, and gender (individually) on post-Recession sleep outcomes in the main analytic and actigraphy samples.

### 2.3 Statistical analysis

All analyses were conducted in R Statistical Software (R Core Team, 2023; https://www.R-project.org/). We first conducted power analyses for the main analytic and actigraphy samples. Next, we examined whether prevalence of Recession hardships and sleep outcomes differed by race and gender, using Welch two sample *t*-tests. Alpha = 0.05 was utilized to determine whether the null hypothesis that the true difference in means between groups was equal to 0 was rejected, or failed to be rejected.

To estimate associations between number of Recession hardships and sleep outcomes, we used regression models. For the global PSQI score, linear regression was used (Li et al., 2021; Xu and Liu, 2021). Recession hardships were included as a predictor of global PSQI scores at post-Recession assessment, and the model controlled for race, gender, age, marital status, education, change in income, and pre-Recession global PSQI score and chronic physical conditions. This model was rerun to assess associations of specific types of Recession hardships (financial, job, and housing hardships) on PSQI. To test moderation by race and gender in the association between number of Recession hardships (overall as well as financial, job, and housing, specifically) and PSQI, an interaction term between race/gender (separately) and Recession hardships was included in the described regression models. Results are presented as unstandardized regression coefficients (B) for these analyses, and multiple *R*^2^ was used to indicate goodness-of-fit of models.

To analyze associations between Recession hardships and actigraphy sleep outcomes, linear regression models were utilized. Recession hardships were included as a predictor of the post-Recession sleep actigraphy mean scores, with consistent covariates as used in the PSQI models (instead of the pre-Recession PSQI score, the corresponding pre-Recession actigraphy measures were used as covariates in the models). We tested associations of types of Recession hardships (financial, job, and housing) and moderation by race and gender on actigraphy sleep outcomes. For actigraphy models, results are reported as unstandardized regression coefficients (B), and multiple *R*^2^ was utilized to indicate goodness-of-fit of models.

Main regression and interaction models used alpha = 0.05 as the threshold for significance. Interaction terms that were significant at alpha = 0.05 were probed using simple slope analysis (Bauer and Curran, 2005). Significant interaction results are presented in figures with 95% confidence intervals illustrated in the graphs.

## 3 Results

### 3.1 Power analysis

For the main analytic sample (*N* = 501), we conducted power analyses to assess effect sizes for post-Recession PSQI between racial (white *n* = 428, Black *n* = 73) and gender (male *n* = 228, female *n* = 273) groups (see Supplementary Table 4 for means and standard deviations of post-Recession sleep outcomes by racial and gender groups). An independent samples *t*-test with 428 participants in Group 1 and 73 participants in Group 2 (Total *N* = 501) indicates that we have 99.67% power to detect a difference of 2.10 or greater in post-Recession global PSQI between white and Black groups (alpha = 0.05, Cohen's *d* = 0.59, two-tailed). This means that we have a 0.33% probability of getting a Type II error, and have high power to detect a difference of 2.10 or greater with medium effect size between racial groups at alpha = 0.05. An independent samples *t*-test with 228 participants in Group 1 and 273 participants in Group 2 (Total *N* = 501) indicates that we have 90.22% power to detect a difference of 0.99 or greater in post-Recession global PSQI between male and female groups (alpha = 0.05, Cohen's *d* = 0.29, two-tailed). This means that we have a 9.78% probability of getting a Type II error, and have high power to detect a difference of 0.99 or greater with small effect size between gender groups at alpha = 0.05.

For the actigraphy sample (*N* = 201), we conducted power analyses to assess effect sizes for post-Recession total sleep time, sleep onset latency, and sleep actigraphy between racial (white *n* = 150, Black *n* = 51) and gender (male *n* = 84, female *n* = 117) groups. An independent samples *t*-test with 150 participants in Group 1 and 51 participants in Group 2 (Total *N* = 201) indicates that we have 96.11% power to detect a difference of 41.06 min or greater in post-Recession total sleep time between white and Black groups (alpha = 0.05, Cohen's *d* = 0.61, two-tailed). This means that we have a 3.89% probability of getting a Type II error, and have high power to detect a difference of 41.06 min or greater with medium effect size between racial groups at alpha = 0.05. An independent samples *t*-test with 84 participants in Group 1 and 117 participants in Group 2 (Total *N* = 201) indicates that we have 96.51% power to detect a difference of 35.37 min or greater in post-Recession total sleep time between male and female groups (alpha = 0.05, Cohen's *d* = 0.54, two-tailed). This means that we have a 3.49% probability of getting a Type II error, and have high power to detect a difference of 35.37 min or greater with medium effect size between gender groups at alpha = 0.05.

An independent samples *t*-test with 150 participants in Group 1 and 51 participants in Group 2 (Total *N* = 201) indicates that we have 99.41% power to detect a difference of 15.52 min or greater in post-Recession sleep onset latency between white and Black groups (alpha = 0.05, Cohen's *d* = 0.73, two-tailed). This means that we have a 0.59% probability of getting a Type II error, and have high power to detect a difference of 15.52 min or greater with medium effect size between racial groups at alpha = 0.05. An independent samples *t*-test with 84 participants in Group 1 and 117 participants in Group 2 (Total *N* = 201) indicates that we have 8.60% power to detect a difference of 1.62 min or greater in post-Recession sleep onset latency between male and female groups (alpha = 0.05, Cohen's *d* = 0.08, two-tailed). This means that we have a 91.40% probability of getting a Type II error, and are underpowered to detect a difference of 1.62 min or greater with small effect size between gender groups at alpha = 0.05.

An independent samples *t*-test with 150 participants in Group 1 and 51 participants in Group 2 (Total *N* = 201) indicates that we have 99.99% power to detect a difference of 8.80% or greater in post-Recession sleep efficiency between white and Black groups (alpha = 0.05, Cohen's *d* = 1.18, two-tailed). This means that we have a 0.01% probability of getting a Type II error, and have high power to detect a difference of 8.80% or greater with large effect size between racial groups at alpha = 0.05. An independent samples *t*-test with 84 participants in Group 1 and 117 participants in Group 2 (Total *N* = 201) indicates that we have 77.92% power to detect a difference of 3.17% or greater in post-Recession sleep efficiency between male and female groups (alpha = 0.05, Cohen's *d* = 0.39, two-tailed). This means that we have a 22.08% probability of getting a Type II error, and have adequate power to detect a difference of 3.17% or greater with medium effect size between gender groups at alpha = 0.05.

### 3.2 Differences by race and gender in recession hardships and sleep

The first set of analyses used Welch two sample *t*-tests to examine whether prevalence of Recession hardships differed by race and gender among the main analytic sample (*N* = 501; see Supplementary Table 3 for breakdowns of Recession hardship prevalence by race and gender). Black participants (mean = 4.05, SD = 3.10, range = 0–13) experienced significantly (*t* = 4.99, df = 88.48, *p* ≤ 0.001) more overall Recession hardships than white participants (mean = 2.15, SD = 2.49, range = 0–13); for financial hardships, Black participants (mean = 2.03, SD = 1.29, range = 0–5) experienced significantly (*t* = 4.74, df = 97.16, *p* ≤ 0.001) more on average than white participants (mean = 1.25, SD = 1.27, range = 0–6); for job-related hardships, Black participants (mean = 0.92, SD = 1.26, range = 0–4) experienced significantly (*t* = 2.85, df = 85.10, *p* ≤ 0.01) more on average compared to white participants (mean = 0.48, SD = 0.90, range = 0–4); and for housing hardships, Black participants (mean = 1.11, SD = 1.48, range = 0–6) experienced significantly (*t* = 3.90, df = 81.57, *p* ≤ 0.001) more on average than white participants (mean = 0.41, SD = 0.91, range = 0–6). Female participants (mean = 2.64, SD = 2.74, range = 0–13) experienced significantly (*t* = 1.98, df = 492.42, *p* ≤ 0.05) more overall Recession hardships than males (mean = 2.17, SD = 2.56, range = 0–13); for financial hardships, females (mean = 1.49, SD = 1.33, range = 0–6) experienced significantly (*t* = 2.43, df = 492.87, *p* ≤ 0.01) more compared to males (mean = 1.21, SD = 1.24, range = 0-5); for job-related hardships, there was not a significant difference (*t* = 0.39, df = 492.80, *p* = 0.347) between females (mean = 0.56, SD = 1.00, range = 0–4) and males (mean = 0.53, SD = 0.94, range = 0–4); and for housing hardships, females (mean = 0.59, SD = 1.08, range = 0–5) experienced significantly (*t* = 1.69, df = 494.64, *p* ≤ 0.05) more on average than males (mean = 0.43, SD = 0.99, range = 0–6).

We repeated descriptive analyses to assess racial and gender group differences in post-Recession sleep outcomes. Supplementary Table 4 illustrates the breakdown of post-Recession sleep variables by race and gender. Black participants (mean = 8.00, SD = 3.77, range = 0–18) had significantly (*t* = 4.48, df = 91.69, *p* ≤ 0.001) higher PSQI scores, indicating poorer global sleep quality, than white participants (mean = 5.90, SD = 3.29, range = 0–18). Females (mean = 6.66, SD = 3.62, range = 0–18) reported significantly (*t* = 3.26, df = 498.35, *p* ≤ 0.001) higher PSQI than males (mean = 5.67, SD = 3.13, range = 0–16). For actigraphy sleep outcomes (*N* = 201), white participants (mean = 407.95, SD = 61.84, range = 173.21–575.38) had significantly (*t* = 3.60, df = 75.81, *p* ≤ 0.001) longer average total sleep time than Black participants (mean = 366.89, SD = 73.07, range = 116.93–494.57); for sleep onset latency, Black participants (mean = 34.33, SD = 25.11, range = 4.21–112.50) had significantly longer (*t* = 4.12, df = 65.39, *p* ≤ 0.001) average times than white participants (mean = 18.81, SD = 16.55, range = 1.86–111.57); and for sleep efficiency, white participants (mean = 86.09, SD = 6.79, range = 56.10–95.82) had significantly higher (*t* = 7.00, df = 75.57, *p* ≤ 0.001) average scores than Black participants (mean = 77.29, SD = 8.06, range = 57.90–91.16). Females (mean = 412.31, SD = 63.55, range = 261.58–575.38) had significantly (*t* = 3.78, df = 173.38, *p* ≤ 0.001) longer average total sleep time than males (mean = 376.94, SD = 66.87, range = 116.93–526.93); for sleep onset latency, there was no significant group difference (*t* = 0.56, df = 173.79, *p* = 0.29) between males (mean = 23.69, SD = 20.77, range = 2.50–111.57) and females (mean = 22.07, SD = 19.82, range = 1.86–112.50); and for sleep efficiency, females (mean = 85.18, SD = 7.22, range = 60.88–95.82) had significantly (*t* = 2.70, df = 155.72, *p* ≤ 0.01) higher average scores compared to males (mean = 82.01, SD = 8.86, range = 56.10–94.25). Thus, the racial differences observed in PSQI were consistently found in actigraphy sleep measures, such that Black participants had significantly worse subjective and objective indicators of sleep than white participants. However, while females reported higher PSQI scores than males, females actually had better actigraphy-measured total sleep time and sleep efficiency than males.

### 3.3 Recession-PSQI associations

Regression results for global PSQI models can be found in Table 2. The first column indicates results using number of overall Recession hardships as a main predictor; the second column examines number of financial hardships as a predictor; the third column examines number of job-related hardships as a predictor; and the final column assesses number of housing hardships as a predictor. A higher number of overall Recession hardships was associated with poorer subjective sleep quality evidenced by higher post-Recession global PSQI scores (B = 0.11, SE = 0.05, *p* ≤ 0.05). This association was found after simultaneously adjusting for sociodemographic covariates, and pre-Recession global PSQI and chronic conditions. Among sub-types of Recession hardships, more financial hardships (B = 0.25, SE = 0.11, *p* ≤ 0.05) and housing hardships (B = 0.27, SE = 0.13, *p* ≤ 0.05) were associated with higher PSQI scores. No significant association was found between job hardships and PSQI (B = 0.01, SE = 0.14, *p* = 0.96). Supplementary Tables 8, 9 rerun these models using dichotomized or binary PSQI scores at pre-Recession and post-Recession assessments; the cut-off used for these analyses was a score of ≤ 5 as having good sleep quality, while a score of >5 as indicative of poor sleep quality (in line with prior research; Buysse et al., 2008; Chai et al., 2023).

**Table 2:** Linear regression models of Recession hardships and MIDUS 3 global PSQI score (*N* = 501).

	**Overall Recession events**		**Financial events**		**Job-related events**		**Housing events**	
	**B**	**95% CI**	**B**	**95% CI**	**B**	**95% CI**	**B**	**95% CI**
**Recession events**	0.11^*^	0.00, 0.21	0.25^*^	0.04, 0.46	0.01	−0.26, 0.27	0.27^*^	0.01, 0.52
**Race (Ref** = **White)**								
Black	1.01^**^	0.27, 1.75	1.03^**^	0.29, 1.76	1.15^**^	0.41, 1.88	1.03^**^	0.29, 1.77
**Gender (Ref** = **Male)**								
Female	0.22	−0.28, 0.73	0.21	−0.29, 0.72	0.22	−0.29, 0.73	0.22	−0.29, 0.72
**Age**	0.00	−0.03, 0.03	0.00	−0.03, 0.03	−0.01	−0.03, 0.02	0.00	−0.03, 0.03
**Marital status (Ref** = **Not married)**								
Married	−0.53^*^	−1.06, 0.00	−0.52^†^	−1.05, 0.00	−0.55^*^	−1.08, −0.02	−0.51^†^	−1.04, 0.02
**Education (Ref** = **HS or less)**								
Some college or more	−0.30	−0.91, 0.31	−0.32	−0.93, 0.29	−0.34	−0.95, 0.27	−0.26	−0.88, 0.35
**Income change (Ref** = **No change)**								
Less now	−0.57^†^	−1.22, 0.08	−0.55^†^	−1.19, 0.09	−0.39	−1.04, 0.25	−0.52	−1.15, 0.12
More now	0.13	−0.50, 0.75	0.13	−0.49, 0.75	0.15	−0.48, 0.77	0.13	−0.49, 0.76
**Global PSQI (M2)**	0.45^***^	0.37, 0.52	0.44^***^	0.37, 0.52	0.46^***^	0.39, 0.54	0.45^***^	0.37, 0.53
**Chronic conditions (M2)**	0.35^***^	0.19, 0.51	0.33^***^	0.17, 0.49	0.36^***^	0.20, 0.51	0.36^***^	0.21, 0.52

Significance level. ^†^p ≤ 0.10, ^*^p ≤ 0.05, ^**^p ≤ 0.01, ^***^p ≤ 0.001.

Overall Recession model: R^2^ = 0.374, F_(10, 490)_ = 29.270, p ≤ 0.001. Financial model: R^2^ = 0.376, F_(10, 490)_ = 29.480, p ≤ 0.001. Job model: R^2^ = 0.369, F_(10, 490)_ = 28.620, p ≤ 0.001. Housing model: R^2^ = 0.374, F_(10, 490)_ = 29.300, p ≤ 0.001.

All parameters listed in table were simultaneously adjusted for.

There were no significant interactions between race and Recession hardships (for overall Recession hardships as well as each of the sub-domains of Recession hardships; overall Recession hardships interacted with race: B = 0.04, SE = 0.12, *p* = 0.72; financial hardships interacted with race: B = 0.35, SE = 0.27, *p* = 0.20; job-related hardships interacted with race: B = −0.16, SE = 0.30, *p* = 0.61; housing hardships interacted with race: B = 0.00, SE = 0.27, *p* = 0.99), or between gender and Recession hardships (overall Recession hardships interacted with gender: B = 0.01, SE = 0.09, *p* = 0.94; financial hardships interacted with gender: B = 0.21, SE = 0.19, *p* = 0.28; job-related hardships interacted with gender: B = −0.23, SE = 0.26, *p* = 0.37; housing hardships interacted with gender: B = −0.09, SE = 0.24, *p* = 0.71), on global PSQI score. The moderation tables for global PSQI can be found in Table 3 (race moderation) and Table 4 (gender moderation).

**Table 3:** Linear regression models of Recession hardships and MIDUS 3 global PSQI score with race interaction (*N* = 501).

	**Overall Recession events**		**Financial events**		**Job–related events**		**Housing events**	
	**B**	**95% CI**	**B**	**95% CI**	**B**	**95% CI**	**B**	**95% CI**
**Recession events**	0.10^†^	−0.02, 0.22	0.20^†^	−0.02, 0.42	0.05	−0.26, 0.36	0.27^†^	−0.04, 0.57
Race ^*^ Recession events	0.04	−0.19, 0.28	0.35	−0.19, 0.89	−0.16	−0.75, 0.44	0.00	−0.53, 0.52
**Race (Ref** = **White)**								
Black	0.86	−0.27, 1.98	0.36	−0.90, 1.62	1.27^**^	0.39, 2.14	1.03^*^	0.16, 1.91
**Gender (Ref** = **Male)**								
Female	0.23	−0.28, 0.74	0.23	−0.27, 0.74	0.21	−0.30, 0.72	0.22	−0.29, 0.72
**Age**	0.00	−0.03, 0.03	0.00	−0.03, 0.03	−0.01	−0.03, 0.02	0.00	−0.03, 0.03
**Marital status (Ref** = **Not married)**								
Married	−0.53^†^	−1.06, 0.00	−0.52^†^	−1.05, 0.01	−0.56^*^	−1.10, −0.03	−0.51^†^	−1.04, 0.02
**Education (Ref** = **HS or less)**								
Some college or more	−0.30	−0.91, 0.31	−0.33	−0.94, 0.27	−0.34	−0.95, 0.27	−0.26	−0.88, 0.35
**Income change (Ref** = **No change)**								
Less now	−0.57^†^	−1.22, 0.08	−0.54^†^	−1.18, 0.09	−0.40	−1.05, 0.25	−0.52	−1.16, 0.12
More now	0.12	−0.50, 0.75	0.11	−0.51, 0.73	0.15	−0.48, 0.78	0.13	−0.49, 0.76
**Global PSQI (M2)**	0.44^***^	0.37, 0.52	0.44^***^	0.36, 0.52	0.46^***^	0.39, 0.54	0.45^***^	0.37, 0.53
**Chronic conditions (M2)**	0.35^***^	0.19, 0.51	0.33^***^	0.17, 0.48	0.35^***^	0.20, 0.51	0.36^***^	0.21, 0.52

Significance level. ^†^p ≤ 0.10, ^*^p ≤ 0.05, ^**^p ≤ 0.01, ^***^p ≤ 0.001.

Overall Recession model: R^2^ = 0.374, F_(11, 489)_ = 26.570, p ≤ 0.001. Financial model: R^2^ = 0.378, F_(11, 489)_ = 26.990, p ≤ 0.001. Job model: R^2^ = 0.369, F_(11, 489)_ = 26.000, p ≤ 0.001. Housing model: R^2^ = 0.374, F_(11, 489)_ = 26.580, p ≤ 0.001.

All parameters listed in table were simultaneously adjusted for.

**Table 4:** Linear regression models of Recession hardships and MIDUS 3 global PSQI score with gender interaction (*N* = 501).

	**Overall Recession events**		**Financial events**		**Job–related events**		**Housing events**	
	**B**	**95% CI**	**B**	**95% CI**	**B**	**95% CI**	**B**	**95% CI**
**Recession events**	0.10	−0.05, 0.25	0.13	−0.17, 0.44	0.14	−0.26, 0.54	0.32^†^	−0.06, 0.70
Gender ^*^ Recession events	0.01	−0.18, 0.19	0.21	−0.17, 0.59	−0.23	−0.74, 0.28	−0.09	−0.56, 0.39
**Race (Ref** = **White)**								
Black	1.02^**^	0.27, 1.76	1.05^**^	0.31, 1.78	1.12^**^	0.38, 1.86	1.02^**^	0.28, 1.76
**Gender (Ref** = **Male)**								
Female	0.20	−0.47, 0.88	−0.06	−0.78, 0.65	0.34	−0.23, 0.92	0.26	−0.30, 0.82
**Age**	0.00	−0.03, 0.03	0.00	−0.03, 0.03	−0.01	−0.03, 0.02	0.00	−0.03, 0.03
**Marital status (Ref** = **Not married)**								
Married	−0.53^†^	−1.06, 0.00	−0.51^†^	−1.04, 0.02	−0.56^*^	−1.09, −0.03	−0.51^†^	−1.04, 0.02
**Education (Ref** = **HS or less)**								
Some college or more	−0.30	−0.91, 0.31	−0.33	−0.94, 0.28	−0.32	−0.93, 0.29	−0.27	−0.88, 0.34
**Income change (Ref** = **No change)**								
Less now	−0.57^†^	−1.22, 0.08	−0.57^†^	−1.21, 0.07	−0.38	−1.02, 0.27	−0.52	−1.15, 0.12
More now	0.13	−0.50, 0.75	0.13	−0.49, 0.76	0.14	−0.49, 0.77	0.13	−0.50, 0.75
**Global PSQI (M2)**	0.45^***^	0.37, 0.52	0.44^***^	0.37, 0.52	0.46^***^	0.39, 0.54	0.45^***^	0.37, 0.53
**Chronic conditions (M2)**	0.35^***^	0.19, 0.51	0.34^***^	0.18, 0.49	0.35^***^	0.20, 0.51	0.36^***^	0.21, 0.52

Significance level. ^†^p ≤ 0.10, ^*^p ≤ 0.05, ^**^p ≤ 0.01, ^***^p ≤ 0.001.

Overall Recession model: R^2^ = 0.374, F_(11, 489)_ = 26.560, p ≤ 0.001. Financial model: R^2^ = 0.377, F_(11, 489)_ = 26.910, p ≤ 0.001. Job model: R^2^ = 0.370, F_(11, 489)_ = 26.080, p ≤ 0.001. Housing model: R^2^ = 0.374, F_(11, 489)_ = 26.600, p ≤ 0.001.

All parameters listed in table were simultaneously adjusted for.

### 3.4 Recession-sleep actigraphy associations

Regression results for total actigraphy sleep time can be found in Table 5. There were no significant associations between overall nor specific Recession hardships on total sleep time (overall Recession hardships: B = −0.64, SE = 1.77, *p* = 0.72; financial hardships: B = −0.03, SE = 3.62, *p* = 0.99; job-related hardships: B = 1.42, SE = 4.46, *p* = 0.75; housing hardships: B = −4.82, SE = 4.17, *p* = 0.25). Moderation analyses revealed no significant interactions between Recession hardships and race (overall Recession hardships interacted with race: B = 1.27, SE = 3.31, *p* = 0.70; financial hardships interacted with race: B = 9.34, SE = 7.04, *p* = 0.19; job-related hardships interacted with race: B = 13.55, SE = 8.63, *p* = 0.12; housing hardships interacted with race: B = −13.99, SE = 7.88, *p* = 0.08) or gender (overall Recession hardships interacted with gender: B = −3.76, SE = 3.30, *p* = 0.26; financial hardships interacted with gender: B = −3.82, SE = 6.37, *p* = 0.55; job-related hardships interacted with gender: B = −11.87, SE = 9.09, *p* = 0.19; housing hardships interacted with gender: B = −7.19, SE = 8.91, *p* = 0.42) on total sleep time (Tables 6, 7).

**Table 5:** Linear regression models of Recession hardships and MIDUS 3 total sleep time (*N* = 201).

	**Overall Recession events**		**Financial events**		**Job–related events**		**Housing events**	
	**B**	**95% CI**	**B**	**95% CI**	**B**	**95% CI**	**B**	**95% CI**
**Recession events**	−0.64	−4.13, 2.84	−0.03	−7.18, 7.12	1.42	−7.38, 10.21	−4.82	−13.04, 3.40
**Race (Ref** = **White)**								
Black	−32.60^**^	−55.23, −10.97	−33.91^**^	−55.53, −12.29	−34.35^**^	−54.88, −13.82	−30.13^**^	−51.45, −8.80
**Gender (Ref** = **Male)**								
Female	26.91^**^	9.36, 44.47	27.03^**^	9.45, 44.60	27.12^**^	9.57, 44.67	27.09^**^	9.60, 44.58
**Age**	0.64	−0.30, 1.57	0.69	−0.26, 1.63	0.71	−0.19, 1.62	0.60	−0.31, 1.50
**Marital status (Ref** = **Not married)**								
Married	−1.82	−19.42, 15.78	−1.71	−19.31, 15.88	−1.68	−19.27, 15.91	−2.58	−20.18, 15.01
**Education (Ref** = **HS or less)**								
Some college or more	−21.05^*^	−39.50, −2.60	−21.26^*^	−39.77, −2.75	−21.30^*^	−39.71, −2.89	−21.03^*^	−39.39, −2.67
**Income change (Ref**=**No change)**								
Less now	14.31	−6.69, 35.32	13.59	−7.18, 34.35	13.02	−7.89, 33.93	15.58	−5.26, 36.41
More now	24.20^*^	2.89, 45.50	24.55^*^	3.19, 45.90	24.63^*^	3.41, 45.85	23.74^*^	2.55, 44.93
**Total sleep time (M2)**	0.39^***^	0.25, 0.52	0.39^***^	0.26, 0.52	0.39^***^	0.26, 0.53	0.40^***^	0.26, 0.53
**Chronic conditions (M2)**	−2.01	−6.49, 2.46	−2.14	−6.69, 2.41	−2.16	−6.58, 2.26	−1.92	−6.34, 2.50

Significance level. ^*^p ≤ 0.05, ^**^p ≤ 0.01, ^***^p ≤ 0.001.

Overall Recession model: R^2^ = 0.325, F_(10, 190)_ = 9.158, p ≤ 0.001. Financial model: R^2^ = 0.325, F_(10, 190)_ = 9.139, p ≤ 0.001. Job model: R^2^ = 0.325, F_(10, 190)_ = 9.154, p ≤ 0.001. Housing model: R^2^ = 0.330, F_(10, 190)_ = 9.337, p ≤ 0.001.

All parameters listed in table were simultaneously adjusted for.

**Table 6:** Linear regression models of Recession hardships and MIDUS 3 total sleep time with race interaction (*N* = 201).

	**Overall Recession events**		**Financial events**		**Job–related events**		**Housing events**	
	**B**	**95% CI**	**B**	**95% CI**	**B**	**95% CI**	**B**	**95% CI**
**Recession events**	−1.15	−5.51, 3.21	−3.18	−11.72, 5.35	−4.67	−16.30, 6.96	2.52	−9.03, 14.06
Race ^*^ Recession events	1.27	−5.26, 7.79	9.34	−4.55, 23.23	13.55	−3.48, 30.58	−13.99^†^	−29.53, 1.56
**Race (Ref** = **White)**								
Black	−36.92^*^	−68.02, −5.81	−51.47^**^	−85.34, −17.60	−42.91^***^	−66.02, −19.80	−18.20	−43.21, 6.81
**Gender (Ref** = **Male)**								
Female	26.91^**^	9.32, 44.50	27.09^**^	9.56, 44.63	27.06^**^	9.58, 44.55	27.09^**^	9.70, 44.48
**Age**	0.63	−0.31, 1.57	0.64	−0.31, 1.58	0.69	−0.22, 1.59	0.54	−0.36, 1.44
**Marital status (Ref** = **Not married)**								
Married	−2.02	−19.69, 15.65	−2.66	−20.28, 14.96	−1.50	−19.03, 16.02	−1.29	−18.85, 16.27
**Education (Ref** = **HS or less)**								
Some college or more	−21.01^*^	−39.50, −2.52	−21.26^*^	−39.74, −2.79	−20.63^*^	−38.99, −2.26	−20.71^*^	−38.97, −2.45
**Income change (Ref** = **No change)**								
Less now	14.58	−6.52, 35.67	13.68	−7.05, 34.40	14.76	−6.19, 35.70	13.90	−6.91, 34.70
More now	24.19^*^	2.84, 45.54	23.78^*^	2.44, 45.13	25.20^*^	4.05, 46.35	23.27^*^	2.20, 44.35
**Total sleep time (M2)**	0.39^***^	0.26, 0.52	0.40^***^	0.26, 0.53	0.40^***^	0.26, 0.53	0.39^***^	0.26, 0.52
**Chronic conditions (M2)**	−1.97	−6.46, 2.53	−2.11	−6.65, 2.43	−1.90	−6.31, 2.52	−2.07	−6.47, 2.33

Significance level. ^†^p ≤ 0.10, ^*^p ≤ 0.05, ^**^p ≤ 0.01, ^***^p ≤ 0.001.

Overall Recession model: R^2^ = 0.326, F_(11, 189)_ = 8.302, p ≤ 0.001. Financial model: R^2^ = 0.331, F_(11, 189)_ = 8.501, p ≤ 0.001. Job model: R^2^ = 0.334, F_(11, 189)_ = 8.609, p ≤ 0.001. Housing model: R^2^ = 0.341, F_(11, 189)_ = 8.870, p ≤ 0.001.

All parameters listed in table were simultaneously adjusted for.

**Table 7:** Linear regression models of Recession hardships and MIDUS 3 total sleep time with gender interaction (*N* = 201).

	**Overall Recession events**		**Financial events**		**Job–related events**		**Housing events**	
	**B**	**95% CI**	**B**	**95% CI**	**B**	**95% CI**	**B**	**95% CI**
**Recession events**	1.96	−3.74, 7.65	2.23	−8.09, 12.54	9.49	−5.53, 24.50	0.60	−14.99, 16.19
Gender ^*^ Recession events	−3.76	−10.26, 2.74	−3.82	−16.39, 8.75	−11.87	−29.80, 6.05	−7.19	−24.76, 10.39
**Race (Ref** = **White)**								
Black	−33.14^**^	−54.77, −11.50	−34.04^**^	−55.70, −12.38	−34.51^**^	−55.01, −14.02	−30.40^**^	−51.76, −9.04
**Gender (Ref** = **Male)**								
Female	35.33^**^	12.53, 58.14	31.98^**^	7.98, 55.98	32.67^**^	13.25, 52.09	30.41^**^	11.12, 49.71
**Age**	0.60	−0.34, 1.54	0.67	−0.28, 1.62	0.67	−0.24, 1.57	0.56	−0.34, 1.47
**Marital status (Ref** = **Not married)**								
Married	−2.43	−20.05, 15.19	−2.23	−19.95, 15.48	−1.64	−19.20, 15.92	−2.68	−20.30, 14.93
**Education (Ref** = **HS or less)**								
Some college or more	−20.92^*^	−39.36, −2.49	−21.29^*^	−39.83, −2.75	−20.38^*^	−38.81, −1.94	−21.12^*^	−39.49, −2.74
**Income change (Ref** = **No change)**								
Less now	15.01	−6.01, 36.03	13.76	−7.05, 34.57	13.23	−7.65, 34.10	16.57	−4.42, 37.57
More now	23.22^*^	1.87, 44.57	23.75^*^	2.20, 45.30	23.56^*^	2.32, 44.80	23.84^*^	2.62, 45.05
**Total sleep time (M2)**	0.40^***^	0.26, 0.53	0.39^***^	0.26, 0.53	0.40^***^	0.27, 0.54	0.40^***^	0.27, 0.53
**Chronic conditions (M2)**	−2.30	−6.81, 2.20	−2.29	−6.87, 2.29	−2.28	−6.70, 2.13	−2.03	−6.47, 2.40

Significance level. ^*^p ≤ 0.05, ^**^p ≤ 0.01, ^***^p ≤ 0.001.

Overall Recession model: R^2^ = 0.330, F_(11, 189)_ = 8.457, p ≤ 0.001. Financial model: R^2^ = 0.326, F_(11, 189)_ = 8.313, p ≤ 0.001. Job model: R^2^ = 0.331, F_(11, 189)_ = 8.508, p ≤ 0.001. Housing model: R^2^ = 0.332, F_(11, 189)_ = 8.531, p ≤ 0.001.

All parameters listed in table were simultaneously adjusted for.

Regression results for sleep onset latency can be found in Table 8. Overall Recession hardships, as well as individual domains of hardships, were not significantly associated with sleep onset latency (overall Recession hardships: B = 0.52, SE = 0.59, *p* = 0.38; financial hardships: B = 1.56, SE = 1.19, *p* = 0.19; job-related hardships: B = −0.87, SE = 1.49, *p* = 0.56; housing hardships: B = 1.50, SE = 1.38, *p* = 0.28). Moderation analyses revealed no significant interactions between Recession hardships and race (overall Recession hardships interacted with race: B = −0.28, SE = 1.09, *p* = 0.80; financial hardships interacted with race: B = 1.21, SE = 2.31, *p* = 0.60; job-related hardships interacted with race: B = −5.31, SE = 2.85, *p* = 0.06; housing hardships interacted with race: B = 1.05, SE = 2.61, *p* = 0.69) or gender (overall Recession hardships interacted with gender: B = 0.92, SE = 1.08, *p* = 0.40; financial hardships interacted with gender: B = 0.90, SE = 2.07, *p* = 0.66; job-related hardships interacted with gender: B = 0.06, SE = 3.04, *p* = 0.98; housing hardships interacted with gender: B = 5.54, SE = 2.89, *p* = 0.06) on sleep onset latency (Tables 9, 10).

**Table 8:** Linear regression models of Recession hardships and MIDUS 3 sleep onset latency (*N* = 201).

	**Overall Recession events**		**Financial events**		**Job–related events**		**Housing events**	
	**B**	**95% CI**	**B**	**95% CI**	**B**	**95% CI**	**B**	**95% CI**
**Recession events**	0.52	−0.65, 1.69	1.56	−0.80, 3.91	−0.87	−3.80, 2.07	1.50	−1.22, 4.22
**Race (Ref** = **White)**								
Black	11.84^***^	4.86, 18.82	11.32^**^	4.32, 18.32	12.98^***^	6.29, 19.68	11.82^***^	4.95, 18.69
**Gender (Ref** = **Male)**				
Female	−3.78	−9.46, 1.89	−3.64	−9.31, 2.03	−3.77	−9.45, 1.92	−3.98	−9.67, 1.71
**Age**	−0.03	−0.34, 0.27	−0.01	−0.31, 0.30	−0.09	−0.38, 0.21	−0.04	−0.34, 0.25
**Marital status (Ref** = **Not married)**				
Married	−0.38	−6.11, 5.34	−0.52	−6.22, 5.19	−0.54	−6.27, 5.18	−0.24	−5.97, 5.49
**Education (Ref** = **HS or less)**				
Some college or more	−4.07	−10.05 1.90	−4.31	−10.29, 1.66	−3.80	−9.77, 2.16	−3.92	−9.87, 2.03
**Income change (Ref** = **No change)**				
Less now	2.52	−4.35, 9.39	2.59	−4.17, 9.35	3.52	−3.33, 10.37	2.53	−4.28, 9.34
More now	−4.43	−11.42, 2.57	−4.19	−11.18, 2.81	−4.59	−11.58, 2.40	−4.43	−11.41, 2.56
**Sleep onset latency (M2)**	0.10^†^	−0.02, 0.22	0.10^†^	−0.01, 0.22	0.12^*^	0.00, 0.24	0.11^†^	−0.01, 0.22
**Chronic conditions (M2)**	1.03	−0.46, 2.52	0.90	−0.61, 2.41	1.09	−0.39, 2.57	1.06	−0.42, 2.54

Significance level. ^†^p < 0.10, ^*^p ≤ 0.05, ^**^p ≤ 0.01, ^***^p ≤ 0.001.

Overall Recession model: R^2^ = 0.203, F_(10, 190)_ = 4.844, p ≤ 0.001. Financial model: R^2^ = 0.207, F_(10, 190)_ = 4.960, p ≤ 0.001. Job model: R^2^ = 0.201, F_(10, 190)_ = 4.789, p ≤ 0.001. Housing model: R^2^ = 0.205, F_(10, 190)_ = 4.895, p ≤ 0.001.

All parameters listed in table were simultaneously adjusted for.

**Table 9:** Linear regression models of Recession hardships and MIDUS 3 sleep onset latency with race interaction (*N* = 201).

	**Overall Recession events**		**Financial events**		**Job–related events**		**Housing events**	
	**B**	**95% CI**	**B**	**95% CI**	**B**	**95% CI**	**B**	**95% CI**
**Recession events**	0.63	−0.80, 2.06	1.15	−1.65, 3.95	1.44	−2.36, 5.24	0.96	−2.84, 4.76
Race ^*^ Recession events	−0.28	−2.44, 1.88	1.21	−3.34, 5.76	−5.31^†^	−10.92, 0.31	1.05	−4.11, 6.21
**Race (Ref** = **White)**								
Black	12.78^*^	2.67, 22.89	9.04	−2.02, 20.11	16.21^***^	8.74, 23.68	10.94^**^	2.81, 19.07
**Gender (Ref** = **Male)**								
Female	−3.77	−9.46, 1.93	−3.63	−9.31, 2.05	−3.62	−9.27, 2.03	−4.00	−9.70, 1.70
**Age**	−0.03	−0.34, 0.28	−0.01	−0.32, 0.30	−0.07	−0.37, 0.22	−0.04	−0.34, 0.26
**Marital status (Ref** = **Not married)**								
Married	−0.35	−6.09, 5.40	−0.62	−6.35, 5.11	−0.67	−6.36, 5.03	−0.33	−6.09, 5.43
**Education (Ref** = **HS or less)**								
Some college or more	−4.07	−10.06, 1.92	−4.34	−10.33, 1.65	−4.00	−9.93, 1.94	−3.95	−9.92, 2.02
**Income change (Ref** = **No change)**								
Less now	2.48	−4.42, 9.37	2.58	−4.19, 9.35	2.93	−3.91, 9.76	2.64	−4.20, 9.49
More now	−4.41	−11.42, 2.61	−4.31	−11.34, 2.71	−4.67	−11.62, 2.28	−4.41	−11.41, 2.59
**Sleep onset latency (M2)**	0.11^†^	−0.01, 0.23	0.10^†^	−0.02, 0.22	0.14^*^	0.02, 0.26	0.10^†^	−0.02, 0.22
**Chronic conditions (M2)**	1.01	−0.48, 2.51	0.91	−0.60, 2.42	0.94	−0.54, 2.42	1.08	−0.40, 2.57

Significance level. ^†^p ≤ 0.10, ^*^p ≤ 0.05, ^**^p ≤ 0.01, ^***^p ≤ 0.001.

Overall Recession model: R^2^ = 0.203, F_(11, 189)_ = 4.388, p ≤ 0.001. Financial model: R^2^ = 0.208, F_(11, 189)_ = 4.517, p ≤ 0.001. Job model: R^2^ = 0.216, F_(11, 189)_ = 4.727, p ≤ 0.001. Housing model: R^2^ = 0.206, F_(11, 189)_ = 4.445, p ≤ 0.001.

All parameters listed in table were simultaneously adjusted for.

**Table 10:** Linear regression models of Recession hardships and MIDUS 3 sleep onset latency with gender interaction (*N* = 201).

	**Overall Recession events**		**Financial events**		**Job–related events**		**Housing events**	
	**B**	**95% CI**	**B**	**95% CI**	**B**	**95% CI**	**B**	**95% CI**
**Recession events**	−0.13	−2.03, 1.78	1.02	−2.35, 4.40	−0.91	−6.03, 4.21	−2.72	−7.84, 2.39
Gender ^*^ Recession events	0.92	−1.21, 3.04	0.90	−3.18, 4.98	0.06	−5.94, 6.06	5.54^†^	−0.16, 11.25
**Race (Ref** = **White)**								
Black	12.00^***^	5.01, 19.00	11.37^**^	4.36, 18.39	12.98^***^	6.27, 19.69	12.09^***^	5.26, 18.92
**Gender (Ref** = **Male)**								
Female	−5.85	−13.29, 1.59	−4.83	−12.67, 3.01	−3.79	−10.09, 2.51	−6.58^*^	−12.83, −0.33
**Age**	−0.02	−0.33, 0.28	0.00	−0.31, 0.31	−0.09	−0.38, 0.21	−0.02	−0.31, 0.28
**Marital status (Ref** = **Not married)**								
Married	−0.28	−6.01, 5.45	−0.41	−6.15, 5.33	−0.55	−6.29, 5.20	−0.23	−5.92, 5.46
**Education (Ref** = **HS or less)**								
Some college or more	−4.04	−10.02, 1.94	−4.29	−10.28, 1.71	−3.81	−9.80, 2.18	−3.77	−9.68, 2.15
**Income change (Ref** = **No change)**								
Less now	2.40	−4.47, 9.28	2.56	−4.21, 9.34	3.52	−3.35, 10.39	1.83	−4.97, 8.63
More now	−4.12	−11.16, 2.91	−3.98	−11.06, 3.09	−4.58	−11.64, 2.47	−4.43	−11.37, 2.50
**Sleep onset latency (M2)**	0.11^†^	−0.01, 0.23	0.11^†^	−0.01, 0.22	0.12^*^	0.00, 0.25	0.11^†^	0.00, 0.23
**Chronic conditions (M2)**	1.09	−0.41, 2.58	0.93	−0.59, 2.45	1.09	−0.39, 2.58	1.14	−0.33, 2.61

Significance level. ^†^p ≤ 0.10, ^*^p ≤ 0.05, ^**^p ≤ 0.01, ^***^p ≤ 0.001.

Overall Recession model: R^2^ = 0.206, F_(11, 189)_ = 4.463, p ≤ 0.001. Financial model: R^2^ = 0.208, F_(11, 189)_ = 4.507, p ≤ 0.001. Job model: R^2^ = 0.201, F_(11, 189)_ = 4.331, p ≤ 0.001. Housing model: R^2^ = 0.220, F_(11, 189)_ = 4.847, p ≤ 0.001.

All parameters listed in table were simultaneously adjusted for.

Regression results for sleep efficiency (measured as a percentage) can be found in Table 11. Overall Recession hardships (B = −0.47, SE = 0.19, *p* ≤ 0.05), financial hardships (B = −0.91, SE = 0.39, *p* ≤ 0.05), and housing hardships (B = −1.54, SE = 0.44, *p* ≤ 0.001) were each negatively associated with sleep efficiency. There was no significant association between job hardships and post-Recession sleep efficiency (B = 0.19, SE = 0.49, *p* = 0.70).

**Table 11:** Linear regression models of Recession hardships and MIDUS 3 sleep efficiency (*N* = 201).

	**Overall Recession events**		**Financial events**		**Job–related events**		**Housing events**	
	**B**	**95% CI**	**B**	**95% CI**	**B**	**95% CI**	**B**	**95% CI**
**Recession events**	−0.47^*^	−0.85, −0.10	−0.91^*^	−1.68, −0.14	0.19	−0.78, 1.16	−1.54^***^	−2.40, −0.67
**Race (Ref** = **White)**								
Black	−4.48^***^	−6.92, −2.04	−4.46^**^	−6.92, −2.00	−5.32^***^	−7.71, −2.93	−4.22^***^	−6.61, −1.82
**Gender (Ref** = **Male)**								
Female	2.46^*^	0.51, 4.40	2.36^*^	0.42, 4.31	2.42^*^	0.45, 4.40	2.57^**^	0.65, 4.49
**Age**	−0.02	−0.12, 0.08	−0.02	−0.13, 0.08	0.02	−0.08, 0.12	−0.01	−0.11, 0.08
**Marital status (Ref** = **Not married)**								
Married	1.07	−0.80, 2.94	1.17	−0.70, 3.04	1.15	−0.75, 3.05	0.90	−0.95, 2.75
**Education (Ref** = **HS or less)**								
Some college or more	0.86	−1.09, 2.81	0.94	−1.02, 2.90	0.69	−1.29, 2.66	0.73	−1.19, 2.64
**Income change (Ref** = **No change)**								
Less now	0.79	−1.48, 3.07	0.54	−1.71, 2.78	0.09	−2.22, 2.40	0.81	−1.40, 3.03
More now	1.65	−0.65, 3.95	1.57	−0.73, 3.88	1.83	−0.50, 4.16	1.58	−0.68, 3.84
**Sleep efficiency (M2)**	0.32^***^	0.21, 0.43	0.33^***^	0.22, 0.43	0.34^***^	0.23, 0.45	0.33^***^	0.23, 0.44
**Chronic conditions (M2)**	−0.27	−0.76, 0.21	−0.23	−0.72, 0.27	−0.35	−0.84, 0.14	−0.29	−0.76, 0.19

Significance level. ^*^p ≤ 0.05, ^**^p ≤ 0.01, ^***^p ≤ 0.001.

Overall Recession model: R^2^ = 0.469, F_(10, 190)_ = 16.780, p ≤ 0.001. Financial model: R^2^ = 0.467, F_(10, 190)_ = 16.670, p ≤ 0.001. Job model: R^2^ = 0.452, F_(10, 190)_ = 15.690, p ≤ 0.001. Housing model: R^2^ = 0.485, F_(10, 190)_ = 17.900, p ≤ 0.001.

All parameters listed in table were simultaneously adjusted for.

**Figure 1:**
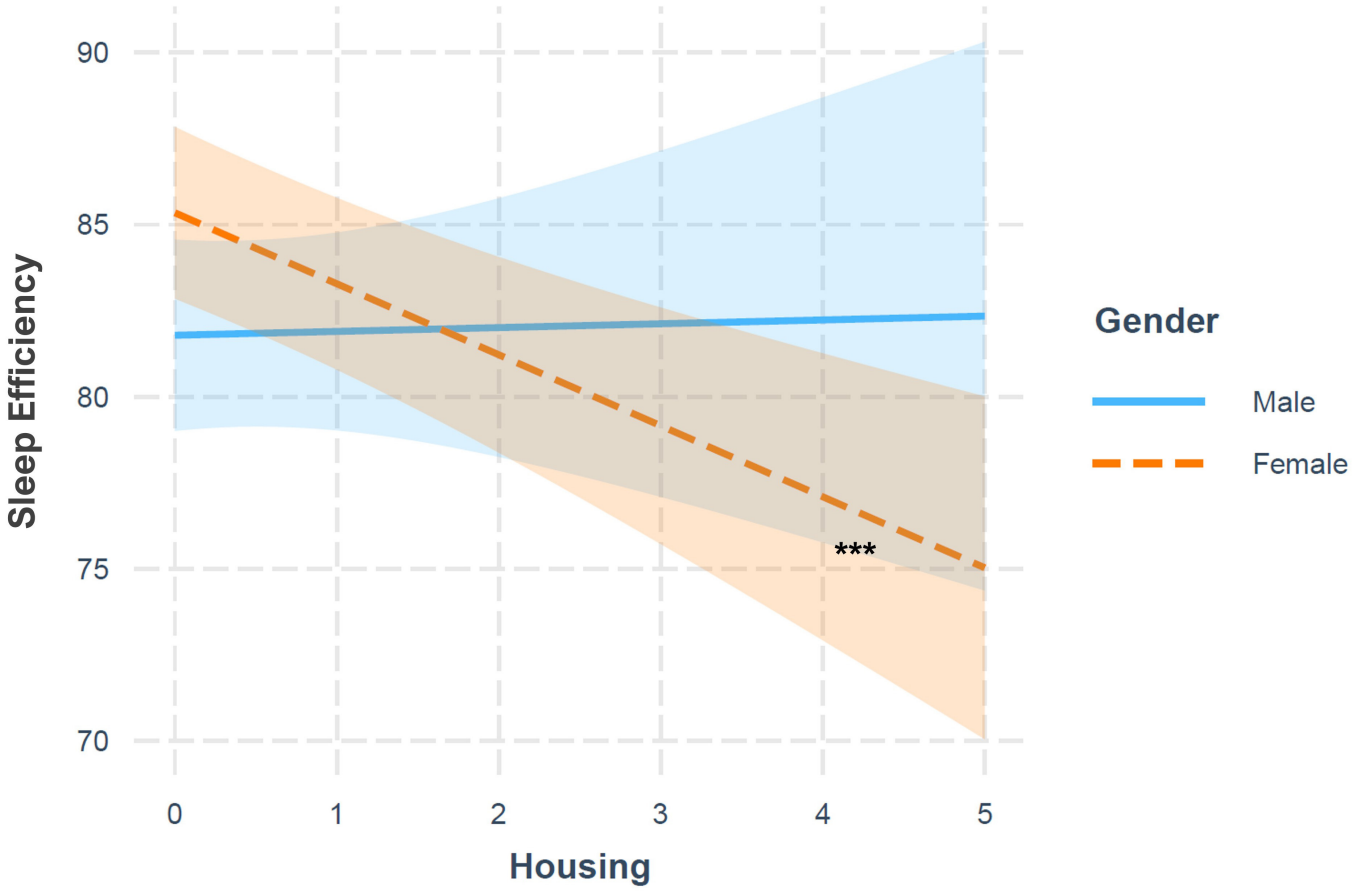
Associations between housing hardships and MIDUS 3 sleep efficiency: moderation by gender (*N* = 201). Significance level. ***p ≤ 0.001.

Moderation analysis for sleep efficiency revealed that overall Recession hardships, financial hardships, and housing hardships did not interact with race to significantly predict sleep efficiency (overall Recession hardships interacted with race: B = 0.39, SE = 0.35, *p* = 0.27; financial hardships interacted with race: B = 0.76, SE = 0.75, *p* = 0.31; housing hardships interacted with race: B = −0.01, SE = 0.84, *p* = 0.99). A significant interaction between job hardships and race was probed (B = 2.02, SE = 0.93, *p* ≤ 0.05; see Table 12). However, simple slope analyses indicated that the interaction effects were not significant at alpha = 0.05 (Black participants' slope estimate = 1.31, SE = 0.71, *p* = 0.07; white participants' slope estimate = −0.72, SE = 0.64, *p* = 0.26). There was a significant interaction result for gender moderation on sleep efficiency (see Table 13). The interaction term for housing hardships and gender (B = −2.17, SE = 0.93, *p* ≤ 0.05) revealed that females who experienced more housing hardships had lower sleep efficiency (slope estimate = −2.06, SE = 0.49, *p* ≤ 0.001) than males (slope estimate = 0.11, SE = 0.83, *p* = 0.89; see Figure 1). However, there were no significant gender interaction effects on sleep efficiency for overall Recession hardships (B = −0.68, SE = 0.35, *p* = 0.051), financial hardships (B = −1.18, SE = 0.67, *p* = 0.08) or job-related hardships (B = −0.73, SE = 0.99, *p* = 0.46).

**Table 12:** Linear regression models of Recession hardships and MIDUS 3 sleep efficiency with race interaction (*N* = 201).

	**Overall Recession events**		**Financial events**		**Job–related events**		**Housing events**	
	**B**	**95% CI**	**B**	**95% CI**	**B**	**95% CI**	**B**	**95% CI**
**Recession events**	−0.63^**^	−1.09, −0.16	−1.17^*^	−2.09, −0.25	−0.72	−1.98, 0.55	−1.53^*^	−2.76, −0.30
Race ^*^ Recession events	0.39	−0.30, 1.08	0.76	−0.72, 2.25	2.02^*^	0.19, 3.86	−0.01	−1.67, 1.64
**Race (Ref** = **White)**								
Black	−5.83^***^	−9.25, −2.41	−5.92^**^	−9.67, −2.17	−6.58^***^	−9.22, −3.95	−4.21^**^	−7.02, −1.39
**Gender (Ref** = **Male)**								
Female	2.48^*^	0.53, 4.42	2.39^*^	0.44, 4.34	2.41^*^	0.45, 4.37	2.57^**^	0.65, 4.49
**Age**	−0.02	−0.12, 0.08	−0.03	−0.13, 0.07	0.01	−0.08, 0.11	−0.01	−0.11, 0.08
**Marital status (Ref** = **Not married)**								
Married	1.01	−0.86, 2.89	1.10	−0.78, 2.98	1.18	−0.70, 3.07	0.90	−0.96, 2.76
**Education (Ref** = **HS or less)**								
Some college or more	0.86	−1.09, 2.81	0.93	−1.03, 2.89	0.77	−1.19, 2.73	0.73	−1.20, 2.65
**Income change (Ref** = **No change)**								
Less now	0.87	−1.41, 3.15	0.54	−1.71, 2.78	0.32	−1.97, 2.62	0.81	−1.42, 3.05
More now	1.64	−0.66, 3.94	1.51	−0.81, 3.82	1.89	−0.42, 4.20	1.58	−0.69, 3.85
**Sleep efficiency (M2)**	0.32^***^	0.21, 0.43	0.33^***^	0.22, 0.43	0.35^***^	0.24, 0.46	0.33^***^	0.23, 0.44
**Chronic conditions (M2)**	−0.26	−0.74, 0.23	−0.22	−0.72, 0.27	−0.31	−0.79, 0.17	−0.29	−0.76, 0.19

Significance level. ^*^p ≤ 0.05, ^**^p ≤ 0.01, ^***^p ≤ 0.001.

Overall Recession model: R^2^ = 0.472, F_(11, 189)_ = 15.390, p ≤ 0.001. Financial model: R^2^ = 0.470, F_(11, 189)_ = 15.250, p ≤ 0.001. Job model: R^2^ = 0.466, F_(11, 189)_ = 14.970, p ≤ 0.001. Housing model: R^2^ = 0.485, F_(11, 189)_ = 16.190, p ≤ 0.001.

All parameters listed in table were simultaneously adjusted for.

**Table 13:** Linear regression models of Recession hardships and MIDUS 3 sleep efficiency with gender interaction (*N* = 201).

	**Overall Recession events**		**Financial events**		**Job–related events**		**Housing events**	
	**B**	**95% CI**	**B**	**95% CI**	**B**	**95% CI**	**B**	**95% CI**
**Recession events**	0.01	−0.60, 0.61	−0.22	−1.31, 0.88	0.69	−0.97, 2.34	0.11	−1.52, 1.74
Gender ^*^ Recession events	−0.68^†^	−1.37, 0.00	−1.18^†^	−2.51, 0.15	−0.73	−2.68, 1.23	−2.17^*^	−4.00, −0.34
**Race (Ref** = **White)**								
Black	−4.55^***^	−6.98, −2.13	−4.52^***^	−6.96, −2.07	−5.31^***^	−7.70, −2.91	−4.26^***^	−6.63, −1.89
**Gender (Ref** = **Male)**								
Female	3.99^**^	1.52, 6.46	3.92^**^	1.31, 6.54	2.75^*^	0.59, 4.92	3.56^***^	1.49, 5.63
**Age**	−0.03	−0.13, 0.07	−0.03	−0.13, 0.07	0.01	−0.08, 0.11	−0.03	−0.12, 0.07
**Marital status (Ref** = **Not married)**								
Married	0.98	−0.88, 2.84	1.03	−0.84, 2.90	1.16	−0.74, 3.06	0.88	−0.94, 2.71
**Education (Ref** = **HS or less)**								
Some college or more	0.85	−1.09, 2.78	0.91	−1.04, 2.86	0.73	−1.25, 2.72	0.68	−1.22, 2.57
**Income change (Ref** = **No change)**								
Less now	0.86	−1.40, 3.12	0.57	−1.67, 2.80	0.08	−2.24, 2.39	1.06	−1.14, 3.27
More now	1.42	−0.88, 3.71	1.31	−1.01, 3.62	1.74	−0.61, 4.09	1.57	−0.67, 3.81
**Sleep efficiency (M2)**	0.33^***^	0.22, 0.44	0.33^***^	0.22, 0.44	0.35^***^	0.24, 0.46	0.34^***^	0.24, 0.45
**Chronic conditions (M2)**	−0.32	−0.80, 0.16	−0.27	−0.76, 0.22	−0.36	−0.85, 0.13	−0.32	−0.79, 0.15

Significance level. ^†^p ≤ 0.10, ^*^p ≤ 0.05, ^**^p ≤ 0.01, ^***^p ≤ 0.001.

Overall Recession model: R^2^ = 0.480, F_(11, 189)_ = 15.830, p ≤ 0.001. Financial model: R^2^ = 0.476, F_(11, 189)_ = 15.590, p ≤ 0.001. Job model: R^2^ = 0.454, F_(11, 189)_ = 14.280, p ≤ 0.001. Housing model: R^2^ = 0.500, F_(11, 189)_ = 17.160, p ≤ 0.001.

All parameters listed in table were simultaneously adjusted for.

## 4 Discussion

This study shows retrospective Recession hardships are associated with 10-year changes in sleep in a national sample of adults. Consistent with the SDoH perspective (Navarro, 2009), Black and female participants were more likely to be exposed to Recession hardships compared to white and male participants, respectively. We also found racial and gender differences in sleep outcomes consistent with extant literature (Dzaja et al., 2005; Krishnan and Collop, 2006; Adenekan et al., 2013; Fuller-Rowell et al., 2016; Grandner et al., 2016; Hale et al., 2020). More importantly, the Recession-sleep link varied by race and gender, which suggests that certain groups may be more susceptible to adverse health impacts of economic hardships. Below we discuss main findings from this study.

Experiencing more overall Recession hardships, as well as a higher number of financial and housing hardships, was associated with poorer global PSQI score and actigraphy sleep efficiency. Job hardships were not significantly associated with any of the sleep measures assessed in the study. It is possible that job hardships experienced in the aftermath of the Recession were more transitory compared to financial impacts (e.g., debt, bankruptcy) or housing impacts (e.g., not being able to afford housing payments, experiencing eviction). Additionally, financial security and housing security may serve more immediate fundamental living needs that are more closely related to sleep compared to job security. For example, living without secure shelter, a basic need, is likely to exacerbate sleep issues by increasing exposure to crowded, noisy conditions, or even environmental toxins (Baker et al., 2017; Swope and Hernández, 2019; Mansour et al., 2022); and financial security is critical to obtaining and keeping secure shelter and maintaining the day-to-day needs of living, such as affording food. Conversely, job-related hardships such as losing work may be more transitional, especially if benefits such as unemployment compensation could be taken advantage of, which historically have far shorter wait times compared to governmental housing assistance programs, which average 2 years, or are even closed to applicants in certain parts of the country (Walter et al., 2014; Fenelon et al., 2017; Kim et al., 2017; Keene et al., 2020). The time period between losing a job and finding a new one may also be shorter, depending on individual circumstances, compared to the time to bounce back from financial or housing crises such as credit card debt or eviction. For example, increased debt and loss of net worth among households impacted by the Recession took years to recover to pre-Recession levels, and for many households never did (Addo and Darity, 2021); and because landlords usually examine rental history of prospective tenants, if there is a record of missed housing payments or prior eviction(s), then tenants are much more likely to have their applications for future housing rejected, which can make mobility into housing security exponentially more difficult, and the more severe a housing insecurity experience is, the longer the housing experience generally will be—this can lead to a trend of “serial forced displacement,” which vulnerable and marginalized groups are especially vulnerable to experiencing (Desmond and Kimbro, 2015; Kang, 2021).

We found no evidence of moderation by race on subjective nor objective post-Recession sleep. This was unexpected, as given the minority poverty hypothesis and compounding of stress when experiencing “double jeopardy” of multiple stressors described in stress process frameworks (Almeida et al., 2011; Pearlin and Bierman, 2013; Ong et al., 2017; Surachman et al., 2020), we had expected that Black participants experiencing Recession hardships would have an exacerbation of poor subjective and objective sleep indicators. Given that only 73 and 51 Black participants were in the main analytic and actigraphy samples, respectively, the limited sample size may have contributed to lack of race moderation results. However, it is also possible that the “double jeopardy” hypothesis did not hold for sleep in the same way it was shown to present for Black adults experiencing housing hardships on chronic physical conditions (Bhat et al., 2022) because sleep may be, in some ways, a modifiable behavior that an individual can attempt to control more easily than the direct physiological dysregulation that contributes to chronic conditions (Piazza et al., 2013; Polsky et al., 2022). Or, it may be that Black participants generally have poorer sleep, potentially partially due to high baseline economic hardship even outside the context of the Recession (Gamaldo et al., 2014; Fuller-Rowell et al., 2016), and thus the effect of increased Recession hardships did not significantly increase sleep problems for the Black participants in the study. Without replication studies in other datasets, it is difficult to ultimately pinpoint why this (lack of) result may have been found, and whether the finding is unique to the MIDUS population or generalizable across studies.

There was some evidence of significant moderation by gender for actigraphy sleep efficiency, but not PSQI or other actigraphy-measured sleep outcomes. While women were more likely to have better actigraphy-measured sleep compared to men, their objectively-measured sleep efficiency suffered with increased housing strain, in line with prior literature that women may have more mental health reactivity to aspects of economic strain compared to men (Glonti et al., 2015). It was notable that gender moderation only existed for housing hardships on sleep efficiency, and not any other domains of hardships, nor overall Recession hardships. There is evidence that women may have poorer health outcomes when exposed to housing hardships compared to men (Vasquez-Vera et al., 2022). It is possible that housing insecurity situations may expose women more to unsafe conditions such as violence compared to men; that gendered socialization may mean that women attribute different meanings to the home than men, such as higher sense of belongingness and ontological security; and that, given women still conduct more household work compared to men, there is a greater sense of responsibility and role identity tied up in the concept of housing; all of which may contribute to greater mental strain and poorer sleep quality related to housing hardships (Hook, 2017; Vasquez-Vera et al., 2022). Other hardships, such as financial or job-related stressors, may not evoke the same gendered sleep response if there is also a partner or other close family member(s) who is contributing to household finances through work. Research indicates men generally report better self-rated health than women, perhaps partly due to gendered socialization, whereby traditional ideals of masculinity result in men being less likely to think they are sick or seek medical attention, and therefore underreport health issues (Read and Gorman, 2011). Conversely, women are more sensitive to health issues and aware of health-promoting behaviors than men; and tend to report poorer health but experience greater longevity than men (which is in line with the disparities in subjective vs. objective sleep for women; Read and Gorman, 2011). Hormonal changes such as menopause and associated symptoms of hot flashes and night sweats may contribute to increased sensitivity to sleep issues among aging women (Shaver and Woods, 2015). Due to men potentially underreporting sleep issues, and women potentially overreporting them, gender differences between economic strain and PSQI may have been muted compared to objective actigraphy outcomes.

Overall, results indicate significant direct associations between Recession hardships, as well as individual domains of financial and housing hardships, on both global PSQI and actigraphy-measured sleep efficiency scores. Results also indicate some evidence of significant gender moderation on the relationship between housing hardships and actigraphy-measured sleep efficiency. This provides an indication that hardships experienced in the unique historical context of the Great Recession affect sleep. Interestingly, the lack of association between Recession hardships and other actigraphy-measured sleep outcomes (e.g., total sleep time and sleep onset latency) may provide some evidence that Recession hardships may mainly affect issues with staying asleep following sleep onset, leading to lower sleep efficiency; rather than affecting the amount of time sleeping or the time participants take to fall asleep initially.

It is important to interpret these results given the context of the sleep measures. In the main analytic sample, post-Recession global PSQI differed significantly across racial (white mean = 5.90, Black mean = 8.00) and gender groups (male mean = 5.67, female mean = 6.66; see Supplementary Table 4). Global PSQI score increased from 5.99 pre-Recession to 6.21 post-Recession, which is consistent with literature indicating older adults score higher on the PSQI (Kim et al., 2021a). Additionally, for post-Recession PSQI, among participants who experienced no Recession hardships the mean score was 5.36, while those who experienced at least one Recession hardship had a mean score of 6.45 on the global PSQI (well above the threshold of 5 which indicates poor sleep; Buysse et al., 2008), indicating that those with Recession hardships did generally experience poorer subjective post-Recession sleep, in line with regression results. The average post-Recession sleep efficiency across the actigraphy sample is 83.86%, ranging from 86.09% for white participants, 77.29% for Black participants, 82.01% for male participants, and 85.18% for female participants (*t*-tests indicated racial and gender group differences to be significant; see Supplementary Table 4), which is the lower limit of normal for midlife and older adults according to the National Sleep Foundation (Ohayon et al., 2017). Thus, there is the possibility of spurious relationships with sleep efficiency, such that other factors that are not captured in the regression models may be contributing to associations with sleep efficiency, or that associations between Recession hardships and sleep efficiency are due to correlation rather than causation, particularly given that MIDUS does not provide temporal information on when Recession hardships were experienced, so it is possible that other events that occurred closer in time to the actigraphy sleep measurement may be partially driving these associations. Given that mean pre-Recession sleep efficiency was 80.03%, while mean post-Recession sleep efficiency was 83.86% (see Table 1), interestingly sleep efficiency actually improved across waves, which is not consistent with the trend that sleep efficiency generally decreases in older adulthood (Didikoglu et al., 2020). However, among participants who experienced no Recession hardships, mean post-Recession sleep efficiency was 87.18%, whereas those who experienced at least one Recession hardship had a mean post-Recession sleep efficiency of 82.87%; which may provide credence to the findings that there is a relationship between Recession hardships and poorer sleep efficiency.

Another contextual consideration is the fact that the difference in post-Recession total sleep time is 41.06 min between white and Black participants, and 35.37 min between female and male participants; although *t*-tests indicated these group differences to be significant (see Supplementary Table 4). Across these groups, average post-Recession total sleep time was between 6.11 and 6.87 h, which is below the recommended threshold of 7–8 h, indicating the sample in general was not getting ideal sleep (Chaput et al., 2018). Total sleep time did increase from pre-Recession (mean = 369.27 min) to post-Recession (mean = 397.53 min; see Table 1), which is again interesting given that total sleep time generally decreases with aging (Espiritu, 2008; Li et al., 2018). However, when comparing post-Recession total sleep time between participants with no Recession hardships (mean = 417.74 min) and those with one or more Recession hardships (mean = 391.53 min), those without hardships had better total sleep times by 26.21 min. Post-Recession sleep onset latency was found to be significantly different between Black and white participants (Black participants had on average 15.52 additional minutes to sleep onset), but not between male and female participants (see Supplementary Table 4). White, male, and female participants were around the threshold of acceptable post-Recession sleep onset latency times of 10–20 min, while Black participants exceeded the acceptable time by 14.33 min (Littner et al., 2005; Gandhi et al., 2021). Sleep onset latency decreased between pre-Recession (mean = 28.46 min) and post-Recession (mean = 22.75; see Table 1), which was also inconsistent with prior literature indicating that sleep onset latency generally increases steadily after 50 years and beyond (Espiritu, 2008; Li et al., 2018). However, compared post-Recession sleep onset latency times between those with no Recession hardships (mean = 16.96 min) and those with at least one Recession hardship (mean = 24.46), those with Recession hardships had on average 7.50 extra minutes to sleep onset. Total time in bed can be calculated by dividing total sleep time by sleep efficiency (percentage score divided by 100). Looking at post-Recession time in bed differences across groups, Black participants had < 1 additional minute in bed compared to white participants; and female participants had ~24 additional minutes in bed compared to males. However, time in bed (which can be calculated from the actigraphy data, but is not generally included as an actigraphy-measured sleep variable in studies, compared to total sleep time, sleep onset latency, and sleep efficiency; Kim et al., 2016; Chung, 2017; Owens et al., 2017; Yip et al., 2021) is not a crucial indicator of sleep health in this study because it is not necessarily representative of either quantity (e.g., total sleep time) nor quality (e.g., sleep onset latency and sleep efficiency) of sleep, which were the indicators this paper was concerned with. For example, participants may “go to bed” early to accommodate time for unwinding activities such as late night meditations or scrolling through social media, which can certainly affect measures such as sleep efficiency, but more commonly used measures of sleep efficiency is likely more appropriate to analyze compared to calculating total time in bed given precedents from other studies and potential issues with equivocating total time in bed directly with either sleep quantity or quality (Amez et al., 2020).

While Recession hardships may not seem to have much of a magnitude of effect on sleep, other studies using the MIDUS dataset have reported similar regression results (when looking at standardized and unstandardized regression coefficients) as indicative of significant associations (at an alpha = 0.05) between independent variables of interest and global PSQI as well as sleep actigraphy measures (Chung, 2017; Owens et al., 2017). In fact, across some of the models in this study (see Tables 2, 5, 8, 11), the association between number of Recession hardships and sleep outcomes is comparable in effect size to the association between some of the other sociodemographic variables used in analyses, such as marital status, or education level, with the sleep outcomes of interest. It is also worth highlighting that, outside of regression results, across all post-Recession subjective and objective sleep measures, when looking at mean differences between no hardship and hardship groups, the group with Recession hardships did have poorer sleep indicators; even though for actigraphy outcomes overall there was improvement between pre- and post-Recession. Additionally, across most of the post-Recession sleep outcomes, there were significant mean-level racial and gender differences which indicate unequal distribution of sleep across the population, even if there were few significant moderation results. Given that objective indicators of total sleep time and sleep efficiency have been shown to predict aspects of the PSQI, it was important to examine both subjective and objective sleep indicators in the context of the Recession (Zitser et al., 2022).

The significant direct and moderation associations on global PSQI and sleep efficiency may be especially notable given that, as number of Recession hardships increases, there is a cumulative effect on the sleep outcomes of interest. While the sleep measures used in the study were only evaluated in the past month (for PSQI) or across 7 consecutive days (for actigraphy measures), these results may be indicative of more persistent sleep disparities which can accumulate across time, resulting in prolonged sleep debt which can contribute to further mental and physical decline, especially among females (Dickinson et al., 2018; Fox et al., 2018; Cabeza De Baca et al., 2019). This also may apply to total sleep time and sleep onset latency; while significant results between Recession hardships and these outcomes were not observed in regressions, even small declines in total sleep time, and increases in sleep onset latency, can contribute to more persistent issues with sleep quantity and quality which can lead to detrimental health; and even fairly small group differences in these measures can be indicative of more persistent sleep troubles which can contribute to further inequitable health between groups in the population (Littner et al., 2005; Brindle et al., 2019).

### 4.1 Strengths and limitations

This study has several strengths. The use of longitudinal data on sleep and retrospective reports on post-Recession experiences allowed us to infer whether retrospective Recession hardships are associated with changes in sleep over time. The MIDUS dataset provides breadth of information on Recession experiences, allowing for use of multiple indicators of Recession strain (e.g., overall number of Recession hardships, and individual domains of Recession hardships), which can be used to compare and contrast how specific types of economic adversity may differentially impact sleep for aging adults. Finally, use of self-report and actigraphy sleep measures provided an opportunity to compare how subjective and objective sleep indicators may be consistent or differ across groups of participants experiencing Recession strain.

Limitations include that MIDUS does not provide information on how many times specific Recession events were experienced and when these events occurred. In this study, we focused on capturing retrospective reports of Recession hardships since the 2007–2009 historical event, yet future studies may want to examine whether more frequent or more proximal macro-level stressors have stronger associations with sleep outcomes. We also had a small sample size for actigraphy sleep outcomes, which may limit the generalizability of our analyses to the greater population, particularly for interaction models. Finally, small sample sizes for other minoritized racial groups in MIDUS limited our racial moderation analyses across broader racial/ethnic categorizations.

## 5 Conclusions and implications

This study found that, across a sample of midlife and older adults, Recession hardships overall, and across financial and housing domains, were associated with poorer subjective and objective sleep as measured by sleep efficiency. The results also suggest that social disparities in sleep may relate to unequal experiences of a historical event by population groups. We found that Black participants and females were more likely to be exposed to Recession hardships compared to white participants and males, respectively; that Black participants experienced poorer subjective and objective sleep indicators compared to white participants; while females reported poorer subjective sleep, but had better objective sleep indicators compared to males; and, finally, that females who experienced housing hardships were disproportionately adversely impacted in terms of objective sleep efficiency compared to males.

These findings indicate a need for targeted interventions for groups who are more vulnerable to experiencing economic hardships, such as minoritized groups and women, to reduce population health disparities caused by sleep inequity. Policies and programs to help the recently unemployed and provide housing assistance could buffer adverse effects of economic hardships on sleep and other health outcomes that are exacerbated by poor sleep (Petrov and Lichstein, 2016; Fenelon et al., 2017). Additionally, increased access to healthcare and telemedicine sleep treatments may improve sleep issues for those facing economic strain (Walter et al., 2014; Simon et al., 2017). Specifically, community-based in-person and telehealth interventions have resulted in measurably improved sleep behavior indicators among aging adults. Interventions that include components of cognitive behavioral therapy (CBT), facilitating high levels of emotional resilience to life stressors, educator-led sessions on sleep hygiene and healthy sleep practices, increased screening and diagnosis services to identify and treat any sleep-related disorders, and use of subjective and actigraphy measured sleep quality and quantity indicators, have shown improvements in sleep among participants (Martin et al., 2017; Smallfield and Molitor, 2018; Rottapel et al., 2020; Billings et al., 2021; Tucker et al., 2021; Bentham and Eaves, 2022). Many of these interventions specifically recruited minoritized and/or economically vulnerable participants for sleep-improvement programs, and interventions generally included multiple sessions, including a screening session followed by weekly follow-ups regarding sleep education and therapy intervention (Rottapel et al., 2020; Billings et al., 2021; Tucker et al., 2021). A more global approach to reducing population sleep disparities includes eliminating de-facto segregation of neighborhoods and promoting diversity, enhancing greenspace, improving air quality, and enhancing neighborhood safety and cohesion to reduce some of the neighborhood-level inequalities that may contribute to poorer sleep health (Billings et al., 2021). While there is no one-size-fits-all approach to how best to alleviate sleep problems for economically vulnerable aging adults, using the results of studies similar to this one and automatically providing resources to participants at risk for poorer sleep health (measured by subjective and/or objective sleep measures) can help spread awareness of the associations between economic and recessionary hardships and sleep, as well as provide resources and information to those most at risk of sleep issues; and participants in sleep intervention studies should be compensated to increase incentivization and compliance with protocol. At a community or neighborhood level, increasing access to sleep-related support groups or clinical sleep services at a low cost (from diagnostic sessions to more prolonged care) may help vulnerable groups such as women and gender and racial/ethnic minorities utilize these services. Additionally, combining individual approaches such as CBT, sleep education sessions, or sleep tracking through phone applications with policy-level changes in terms of reducing severity of economic and recessionary hardships through increased access to services and improved neighborhood quality may be effective at tackling sleep disparities at both individual and population levels.

This research also has implications for understanding how other recession experiences, such as those experienced during the COVID-19 pandemic, may affect subjective and objective indicators of sleep for aging adults (Simonelli et al., 2021; Gaston et al., 2023). Thus, healthcare practitioners and aging researchers must be prepared to address deleterious health effects caused by sleep problems for the aging population related to economic and recessionary hardships (Bierman, 2021). Finally, future empirical work should further elucidate the mechanistic pathways that connect stress associated with recession hardships with health behaviors such as sleep, including through pathways of psychological distress and mental health.

## Data Availability

The dataset used for this study is available through the Midlife in the United States Colectica Portal (https://midus.colectica.org/), and analysis code is available upon request from the first author.
