# Supplementary material for: Associations between Recession hardships and subjective and objective sleep measures in the midlife in the United States study: race and gender differences
Source: Front Sleep. 2024 Oct 30;3:1403818. doi: 10.3389/frsle.2024.1403818 (PMC11580659; doi:10.3389/frsle.2024.1403818)
Supplement: Supplementary file 1 [file Table_1.DOCX]

Supplementary Material

# Supplementary Tables

**Table A.1.** Attrition from MIDUS 2 to MIDUS 3 Biomarker Projects

| **Predictors** |  | OR |  | 95% CI |
| --- | --- | --- | --- | --- |
| **Race (M2; Ref=White)** |  |  |  |  |
| Black |  | 0.84 |  | 0.60, 1.18 |
| **Gender (M2; Ref=Male)** |  |  |  |  |
| Female |  | 0.97 |  | 0.76, 1.26 |
| **Age (M2; Ref<65)** |  |  |  |  |
| 65+ |  | 0.29*** |  | 0.22, 0.39 |
| **Chronic Conditions (M2)** |  | 0.88*** |  | 0.82, 0.95 |
| **PSQI Global Score (M2)** |  | 0.98 |  | 0.94, 1.02 |

Significance level. † p ≤ 0.10, * p ≤ 0.05, ** p ≤ 0.01, *** p ≤ 0.001

Model R^2^ = 0.066, X^2^(5, *N* = 1255) = 101.515, p ≤ 0.001

Note. Participants were coded dichotomously (0=not retained, 1=retained), logistic regression used to predict whether participants were retained versus not. All predictors listed in table were simultaneously tested in model. 54.74% of sample retained between MIDUS 2 and MIDUS 3 Biomarker Projects, while 568 individuals were lost.

**Table A.2.** Exclusions in main analytic sample due to selection

| **Sample Size Prior to Exclusions: 687** | |
| --- | --- |
| **Variable** | **N Ineligible (race other than Black/white, NA, missing data responses)** |
| Race (M3) | 51 |
| Income Change (M3) | 64 |
| Chronic Conditions (M2) | 6 |
| Global PSQI (M2) | 44 |
| Global PSQI (M3) | 49 |
| **Total N Excluded: 186**  **Final Sample Size: 501** | |

**Table A.3.** Prevalence of Recession hardships across all items in main analytic sample (*N* = 501) and race and gender breakdowns

| **Recession Impact by Domain** | **Main Sample**  **(*N* = 501)** | **Race** | | **Gender** | |
| --- | --- | --- | --- | --- | --- |
|  |  | **White (*n* = 428)** | **Black (*n* = 73)** | **Male (*n* = 228)** | **Female (*n* = 273)** |
| **Financial hardships** |  |  |  |  |  |
| Declared bankruptcy | 13 (2.6%) | 6 (1.4%) | 7 (9.6%)* | 9 (3.9%)* | 4 (1.5%) |
| Missed a credit card payment | 63 (12.6%) | 45 (10.5%) | 18 (24.7%)* | 23 (10.1%) | 40 (14.7%) |
| Missed other debt payments, car/student loans | 45 (9.0%) | 28 (6.5%) | 17 (23.3%)* | 19 (8.3%) | 26 (9.5%) |
| Increased credit card debt | 119 (23.8%) | 101 (23.6%) | 18 (24.7%) | 46 (20.2%) | 73 (26.7%)* |
| Sold possessions to make ends meet | 75 (15.0%) | 63 (14.7%) | 12 (16.4%) | 27 (11.8%) | 48 (17.6%)* |
| Cut back on spending | 326 (65.1%) | 268 (62.6%) | 58 (79.5%)* | 134 (58.8%) | 192 (70.3%)* |
| Exhausted unemployment benefits | 44 (8.8%) | 26 (6.1%) | 18 (24.7%)* | 19 (8.3%) | 25 (9.2%) |
| **At least one financial hardship** | **353 (70.5%)** | **289 (67.5%)** | **64 (87.7%)*** | **148 (64.9%)** | **205 (75.1%)*** |
| **Job-related hardships** |  |  |  |  |  |
| Lost a job | 81 (16.2%) | 61 (14.3%) | 20 (27.4%)* | 38 (16.7%) | 43 (15.8%) |
| Started a new job did not like it | 43 (8.6%) | 32 (7.5%) | 11 (15.1%)* | 19 (8.3%) | 24 (8.8%) |
| Took job below education/experience | 75 (15.0%) | 53 (12.4%) | 22 (30.1%)* | 33 (14.5%) | 42 (15.4%) |
| Took on an additional job | 74 (14.8%) | 60 (14.0%) | 14 (19.2%) | 30 (13.2%) | 44 (16.1%) |
| **At least one job-related hardship** | **157 (31.3%)** | **125 (29.2%)** | **32 (43.8%)*** | **71 (31.1%)** | **86 (31.5%)** |
| **Housing hardships** |  |  |  |  |  |
| Missed a mortgage or rent payment | 51 (10.2%) | 34 (7.9%) | 17 (23.3%)* | 19 (8.3%) | 32 (11.7%) |
| Was threatened with foreclosure or eviction | 39 (7.8%) | 24 (5.6%) | 15 (20.5%)* | 16 (7.0%) | 23 (8.4%) |
| Sold a home for less than it cost | 27 (5.4%) | 20 (4.7%) | 7 (9.6%) | 11 (4.8%) | 16 (5.9%) |
| Lost a home to foreclosure | 13 (2.6%) | 6 (1.4%) | 7 (9.6%)* | 8 (3.5%) | 5 (1.8%) |
| Lost a home to something other than foreclosure | 13 (2.6%) | 12 (2.8%) | 1 (1.4%) | 3 (1.3%) | 10 (3.7%)* |
| Family/friends moved in to save money | 82 (16.4%) | 61 (14.3%) | 21 (28.8%)* | 26 (11.4%) | 56 (20.5%)* |
| Moved in with family/friends to save money | 33 (6.6%) | 20 (4.7%) | 13 (17.8%)* | 15 (6.6%) | 18 (6.6%) |
| **At least one housing-related hardship** | **138 (27.5%)** | **102 (23.8%)** | **36 (49.3%)*** | **52 (22.8%)** | **86 (31.5%)*** |
| **At least one Recession hardship** | **388 (77.5%)** | **320 (74.8%)** | **68 (93.2%)*** | **166 (72.8%)** | **222 (81.3%)*** |

Note. Values presented as No. (%); *indicates group has greater prevalence of hardship at p ≤ 0.05 using t-test

**Table A.4.** Prevalence of MIDUS 3 sleep outcomes in main analytic sample (*N* = 501) and actigraphy sample (*N* = 201) and race and gender breakdowns

| **Subjective Sleep Outcome (M3)** | **Main Sample**  **(*N* = 501)** | **Race** | | **Gender** | |
| --- | --- | --- | --- | --- | --- |
|  |  | **White (*n* = 428)** | **Black (*n* = 73)** | **Male (*n* = 228)** | **Female (*n* = 273)** |
| **Global PSQI** | **6.21 (3.44)** | **5.90 (3.29)** | **8.00 (3.77)*** | **5.67 (3.13)** | **6.66 (3.62)*** |
| Subjective sleep quality | 1.01 (0.65) | 0.98 (0.64) | 1.22 (0.65)* | 0.95 (0.61) | 1.07 (0.67)* |
| Sleep latency | 1.02 (0.90) | 0.95 (0.88) | 1.40 (0.92)* | 0.81 (0.81) | 1.19 (0.94)* |
| Sleep duration | 0.79 (0.79) | 0.71 (0.73) | 1.26 (0.93)* | 0.86 (0.80)* | 0.73 (0.77) |
| Habitual sleep efficiency | 0.62 (0.92) | 0.52 (0.84) | 1.19 (1.14)* | 0.54 (0.87) | 0.69 (0.95)* |
| Sleep disturbance | 1.29 (0.57) | 1.28 (0.53) | 1.40 (0.74) | 1.20 (0.52) | 1.37 (0.60)* |
| Use of sleeping medication | 0.65 (1.12) | 0.66 (1.12) | 0.63 (1.11) | 0.48 (0.99) | 0.80 (1.20)* |
| Daytime dysfunction | 0.83 (0.63) | 0.81 (0.60) | 0.90 (0.78) | 0.84 (0.62) | 0.81 (0.65) |
| **Actigraphy Sleep Outcome (M3)** | **Actigraphy Sample**  **(*N* = 201)** | **Race** | | **Gender** | |
|  |  | **White (*n* = 150)** | **Black (*n* = 51)** | **Male (*n* = 84)** | **Female (*n* = 117)** |
| **Total Sleep Time (minutes)** | **397.53 (67.12)** | **407.95 (61.84)*** | **366.89 (73.07)** | **376.94 (66.87)** | **412.31 (63.55)*** |
| **Sleep Onset Latency (minutes)** | **22.75 (20.19)** | **18.81 (16.55)** | **34.33 (25.11)*** | **23.69 (20.77)** | **22.07 (19.82)** |
| **Sleep Efficiency (%)** | **83.86 (8.08)** | **86.09 (6.79)*** | **77.29 (8.06)** | **82.01 (8.86)** | **85.18 (7.22)*** |

Note. Values presented as Mean (SD); *indicates group has greater mean of sleep score at p ≤ 0.05 using t-test

**Table A.5.** Percentage of People Reporting Chronic Health Conditions at MIDUS 2 in main analytic sample (*N* = 501)

| **Type of Chronic Condition** |  | **MIDUS 2 % of sample** |
| --- | --- | --- |
| Autoimmune Disorders |  | 1.2 |
| Cancer |  | 8.4 |
| *Cardiovascular Conditions |  | 31.3 |
| Diabetes or High Blood Sugar |  | 6.8 |
| *Digestive Conditions |  | 20.0 |
| Foot Trouble |  | 8.0 |
| Hay Fever |  | 13.4 |
| Gall Bladder Trouble |  | 1.4 |
| *Lung Conditions |  | 11.2 |
| Neurological Conditions |  | 2.8 |
| *Pain-Related Conditions |  | 36.1 |
| Skin Trouble |  | 8.4 |
| Thyroid Disease |  | 6.0 |
| *Trouble with Gums, Mouth, or Teeth |  | 8.2 |
| Urinary or Bladder Problems |  | 8.2 |
| Anxiety/Depression |  | 19.0 |
| **At Least 1 Chronic Condition** |  | **77.8** |

*Combined categories

Cardiovascular conditions = heart disease; stroke; high blood pressure or hypertension

Digestive Conditions = recurring stomach trouble, indigestion, or diarrhea; constipated all/most of time; ulcer

Lung conditions = asthma, bronchitis, emphysema; tuberculosis; other lung problems

Pain-related conditions = arthritis, rheumatism, or other bone/joint diseases; sciatica, lumbago or recurring backache; migraine headaches

Trouble with gums, mouth or teeth = persistent trouble with gums or mouth; persistent trouble with teeth

**Table A.6.** Bivariate correlations of dichotomized and discrete numerical study variables for main analytic sample (*N* = 501)

|  | Race (M3) | Gender (M3) | Age (M3) | Marital Status (M3) | Education (M3) | Chronic Conditions (M2) | Global PSQI (M2) | Global PSQI (M3) | Recession Hardships (M3) |
| --- | --- | --- | --- | --- | --- | --- | --- | --- | --- |
| 1. Race (M3; 1=Black) |  |  |  |  |  |  |  |  |  |
| 2. Gender (M3; 1=Female) | .06 |  |  |  |  |  |  |  |  |
| 3. Age (M3) | -.11** | -.12** |  |  |  |  |  |  |  |
| 4. Marital Status (M3; 1=Married) | -.29*** | -.17*** | .04 |  |  |  |  |  |  |
| 5. Education (M3; 1=Some College +) | -.11** | -.08† | -.02 | .01 |  |  |  |  |  |
| 6. Chronic Conditions (M2) | -.02 | .17*** | .12** | -.07 | -.01 |  |  |  |  |
| 7. Global PSQI (M2) | .15*** | .13*** | -.08† | -.17*** | -.03 | .31*** |  |  |  |
| 8. Global PSQI (M3) | .22*** | .14*** | -.06 | -.21*** | -.07 | .32*** | .56*** |  |  |
| 9. Recession Hardships (M3) | .25*** | .09* | -.26*** | -.14*** | -.09* | .09* | .27*** | .24*** |  |

Significance level. † p < 0.10, *p ≤ 0.05, **p ≤ 0.01, ***p ≤ 0.001

**Table A.7.** Univariable associations using linear regression between Recession hardships, demographic variables, on MIDUS 3 sleep measures in main analytic sample (*N* = 501) and actigraphy sample (*N* = 201)

|  | **Global PSQI** | |  | **Total Sleep Time** | |  | **Sleep Onset Latency** | |  | **Sleep Efficiency** | |
| --- | --- | --- | --- | --- | --- | --- | --- | --- | --- | --- | --- |
|  | B | 95% CI |  | B | 95% CI |  | B | 95% CI |  | B | 95% CI |
| **1. Recession Events** | 0.30*** | 0.19, 0.41 |  | -3.84* | -7.24, -0.45 |  | 1.91*** | 0.91, 2.90 |  | -1.06*** | -1.45, -0.67 |
| **2. Race (Ref=White)** |  |  |  |  |  |  |  |  |  |  |  |
| Black | 2.10*** | 1.26, 2.93 |  | -41.05*** | -61.78, -20.32 |  | 15.52*** | 9.43, 21.62 |  | -8.80*** | -11.08, -6.53 |
| **3. Gender (Ref=Male)** |  |  |  |  |  |  |  |  |  |  |  |
| Female | 0.98** | 0.38, 1.59 |  | 35.38*** | 17.06, 53.70 |  | -1.62 | -7.32, 4.08 |  | 3.17** | 0.93, 5.41 |

Significance level. † p < 0.10, * p < 0.05, ** p < 0.01, *** p < 0.001

1. Recession Models: Global PSQI R^2^ = 0.056, F(1, 499) = 29.420, p ≤ 0.001. Total Sleep Time R^2^ = 0.024, F(1, 199) = 4.977, p ≤ 0.05. Sleep Onset Latency R^2^ = 0.067, F(1, 199) = 14.150, p ≤ 0.001. Sleep Efficiency R^2^ = 0.128, F(1, 199) = 29.250, p ≤ 0.001.

2. Race Models: Global PSQI R^2^ = 0.046, F(1, 499) = 24.270, p ≤ 0.001. Total Sleep Time R^2^ = 0.071, F(1, 199) = 15.250, p ≤ 0.001. Sleep Onset Latency R^2^ = 0.113, F(1, 199) = 25.240, p ≤ 0.001. Sleep Efficiency R^2^ = 0.226, F(1, 199) = 58.040, p ≤ 0.001.

3. Gender Models: Global PSQI R^2^ = 0.020, F(1, 499) = 10.370, p ≤ 0.01. Total Sleep Time R^2^ = 0.068, F(1, 199) = 14.500, p ≤ 0.001. Sleep Onset Latency R^2^ = 0.002, F(1, 199) = 0.314, p = 0.576. Sleep Efficiency R^2^ = 0.038, F(1, 199) = 7.780, p ≤ 0.01.

Note. Recession hardships, demographic variables were independently tested to assess univariable associations with MIDUS 3 sleep measures

**Table A.8.** Linear regression models of Recession hardships and MIDUS 3 global PSQI score, controlling for MIDUS 2 binary PSQI

(*N* = 501)

|  | **Overall Recession Events** | |  | **Financial Events** | |  | **Job-Related Events** | |  | **Housing Events** | |
| --- | --- | --- | --- | --- | --- | --- | --- | --- | --- | --- | --- |
|  | B | 95% CI |  | B | 95% CI |  | B | 95% CI |  | B | 95% CI |
| **Recession Events** | 0.12* | 0.01, 0.23 |  | 0.29* | 0.07, 0.51 |  | -0.02 | -0.30, 0.27 |  | 0.30* | 0.04, 0.57 |
| **Race (Ref=White)** |  |  |  |  |  |  |  |  |  |  |  |
| Black | 1.13** | 0.35, 1.90 |  | 1.14** | 0.37, 1.91 |  | 1.28** | 0.51, 2.05 |  | 1.14** | 0.37, 1.91 |
| **Gender (Ref=Male)** |  |  |  |  |  |  |  |  |  |  |  |
| Female | 0.14 | -0.39, 0.67 |  | 0.13 | -0.40, 0.66 |  | 0.13 | -0.41, 0.66 |  | 0.13 | -0.40, 0.66 |
| **Age** | -0.01 | -0.03, 0.02 |  | 0.00 | -0.03, 0.02 |  | -0.01 | -0.04, 0.01 |  | -0.01 | -0.04, 0.02 |
| **Marital Status (Ref=Not married)** |  |  |  |  |  |  |  |  |  |  |  |
| Married | -0.62* | -1.17, -0.07 |  | -0.61* | -1.16, -0.06 |  | -0.65* | -1.20, -0.09 |  | -0.60* | -1.15, -0.04 |
| **Education (Ref=HS or less)** |  |  |  |  |  |  |  |  |  |  |  |
| Some college or more | -0.37 | -1.01, 0.26 |  | -0.40 | -1.03, 0.24 |  | -0.43 | -1.07, 0.21 |  | -0.34 | -0.98, 0.30 |
| **Income Change (Ref=No change)** |  |  |  |  |  |  |  |  |  |  |  |
| Less Now | -0.62† | -1.30, 0.06 |  | -0.60† | -1.27, 0.07 |  | -0.40 | -1.08, 0.28 |  | -0.56 | -1.23, 0.11 |
| More Now | 0.02 | -0.63, 0.67 |  | -0.02 | -0.63, 0.68 |  | 0.04 | -0.62, 0.70 |  | 0.03 | -0.63, 0.68 |
| **Binary PSQI (M2; Ref≤5)** |  |  |  |  |  |  |  |  |  |  |  |
| >5 | 2.43*** | 1.88, 2.98 |  | 2.41*** | 1.86, 2.95 |  | 2.57*** | 2.03, 3.11 |  | 2.47*** | 1.93, 3.01 |
| **Chronic Conditions (M2)** | 0.48*** | 0.32, 0.64 |  | 0.46*** | 0.30, 0.62 |  | 0.49*** | 0.33, 0.65 |  | 0.49*** | 0.33, 0.65 |

Significance level. † p < 0.10, * p < 0.05, ** p < 0.01, *** p < 0.001

Overall Recession Model: R^2^ = 0.311, F(10, 490) = 22.150, p ≤ 0.001. Financial Model: R^2^ = 0.314, F(10, 490) = 22.430, p ≤ 0.001. Job Model: R^2^ = 0.305, F(10, 490) = 21.490, p ≤ 0.001. Housing Model: R^2^ = 0.312, F(10, 490) = 22.200, p ≤ 0.001.

Note. All parameters listed in table were simultaneously adjusted for

**Table A.9.** Logistic regression models of Recession hardships and MIDUS 3 binary PSQI, controlling for MIDUS 2 binary PSQI (*N* = 501)

|  | **Overall Recession Events** | |  | **Financial Events** | |  | **Job-Related Events** | |  | **Housing Events** | |
| --- | --- | --- | --- | --- | --- | --- | --- | --- | --- | --- | --- |
|  | OR | 95% CI |  | OR | 95% CI |  | OR | 95% CI |  | OR | 95% CI |
| **Recession Events** | 1.03 | 0.94, 1.13 |  | 1.04 | 0.87, 1.24 |  | 1.07 | 0.85, 1.34 |  | 1.08 | 0.87, 1.35 |
| **Race (Ref=White)** |  |  |  |  |  |  |  |  |  |  |  |
| Black | 2.03* | 1.08, 3.92 |  | 2.06* | 1.10, 3.97 |  | 2.06* | 1.10, 3.96 |  | 2.04* | 1.08, 3.92 |
| **Gender (Ref=Male)** |  |  |  |  |  |  |  |  |  |  |  |
| Female | 1.01 | 0.66, 1.54 |  | 1.01 | 0.66, 1.54 |  | 1.01 | 0.66, 1.54 |  | 1.01 | 0.66, 1.54 |
| **Age** | 0.99 | 0.96, 1.01 |  | 0.98 | 0.96, 1.01 |  | 0.98 | 0.96, 1.01 |  | 0.98 | 0.96, 1.01 |
| **Marital Status (Ref=Not married)** |  |  |  |  |  |  |  |  |  |  |  |
| Married | 0.67† | 0.43, 1.04 |  | 0.67† | 0.43, 1.04 |  | 0.66† | 0.43, 1.03 |  | 0.67† | 0.43, 1.04 |
| **Education (Ref=HS or less)** |  |  |  |  |  |  |  |  |  |  |  |
| Some college or more | 0.60† | 0.36, 1.01 |  | 0.60* | 0.36, 0.99 |  | 0.60* | 0.36, 0.99 |  | 0.61† | 0.36, 1.02 |
| **Income Change (Ref=No change)** |  |  |  |  |  |  |  |  |  |  |  |
| Less Now | 0.51* | 0.29, 0.89 |  | 0.53* | 0.31, 0.91 |  | 0.52* | 0.30, 0.90 |  | 0.52* | 0.30, 0.90 |
| More Now | 0.87 | 0.51, 1.47 |  | 0.87 | 0.51, 1.48 |  | 0.87 | 0.51, 1.47 |  | 0.87 | 0.51, 1.47 |
| **Binary PSQI (M2; Ref≤5)** |  |  |  |  |  |  |  |  |  |  |  |
| >5 | 5.64*** | 3.71, 8.70 |  | 5.72*** | 3.76, 8.82 |  | 5.73*** | 3.79, 8.78 |  | 5.71*** | 3.77, 8.75 |
| **Chronic Conditions (M2)** | 1.27*** | 1.11, 1.47 |  | 1.27*** | 1.11, 1.47 |  | 1.28*** | 1.12, 1.47 |  | 1.28*** | 1.11, 1.47 |

Significance level. † p < 0.10, * p < 0.05, ** p < 0.01, *** p < 0.001

Overall Recession Model: R^2^ = 0.200, X^2^(10, *N* = 501) = 138.919, p ≤ 0.001. Financial Model: R^2^ = 0.200, X^2^(10, *N* = 501) = 138.609, p ≤ 0.001. Job Model: R^2^ = 0.200, X^2^(10, *N* = 501) = 138.775, p ≤ 0.001. Housing Model: R^2^ = 0.200, X^2^(10, *N* = 501) = 138.891, p ≤ 0.001.

Note. All parameters listed in table were simultaneously adjusted for

**
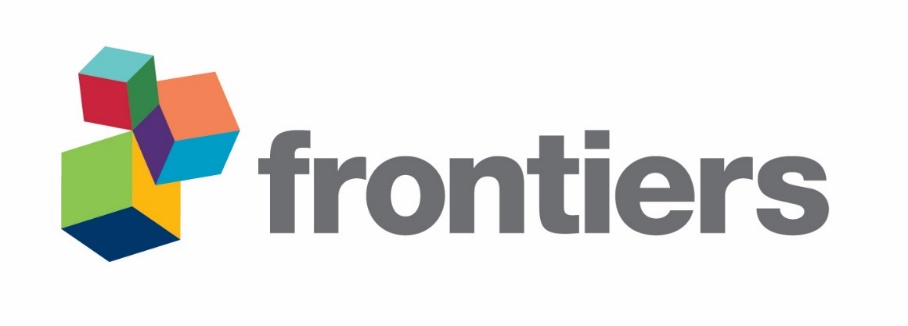
**
